# L-Arginine/Nitric Oxide Pathway Is Altered in Colorectal Cancer and Can Be Modulated by Novel Derivatives from Oxicam Class of Non-Steroidal Anti-Inflammatory Drugs

**DOI:** 10.3390/cancers12092594

**Published:** 2020-09-11

**Authors:** Małgorzata Krzystek-Korpacka, Berenika Szczęśniak-Sięga, Izabela Szczuka, Paulina Fortuna, Marek Zawadzki, Agnieszka Kubiak, Magdalena Mierzchała-Pasierb, Mariusz G. Fleszar, Łukasz Lewandowski, Paweł Serek, Natalia Jamrozik, Katarzyna Neubauer, Jerzy Wiśniewski, Radosław Kempiński, Wojciech Witkiewicz, Iwona Bednarz-Misa

**Affiliations:** 1 Department of Medical Biochemistry, Wroclaw Medical University, 50-368 Wroclaw, Poland; izabela.szczuka@umed.wroc.pl (I.S.); paulina.fortuna@umed.wroc.pl (P.F.); a.kubiak@umed.wroc.pl (A.K.); magdalena.mierzchala-pasierb@umed.wroc.pl (M.M.-P.); fleszar.mariusz@gmail.com (M.G.F.); lukasz.lewandowski@umed.wroc.pl (Ł.L.); pawel.serek@umed.wroc.pl (P.S.); natalia.jamrozik@student.umed.wroc.pl (N.J.); jerzy.wisniewski@umed.wroc.pl (J.W.); iwona.bednarz-misa@umed.wroc.pl (I.B.-M.); 2 Department of Medicinal Chemistry, Faculty of Pharmacy, Wroclaw Medical University, 50-556 Wroclaw, Poland; berenika.szczesniak-siega@umed.wroc.pl; 3 Department of Oncological Surgery, Regional Specialist Hospital, 51-124 Wroclaw, Poland; zawadzki@wssk.wroc.pl (M.Z.); witkiewicz@wssk.wroc.pl (W.W.); 4 Department of Physiotherapy, Wroclaw Medical University, 51-618 Wroclaw, Poland; 5 Department of Gastroenterology and Hepatology, Wroclaw Medical University, 50-556 Wroclaw, Poland; katarzyna.neubauer@umed.wroc.pl (K.N.); radoslaw.kempinski@umed.wroc.pl (R.K.); 6 Research and Development Centre at Regional Specialist Hospital, 51-124 Wroclaw, Poland

**Keywords:** arginase (ARG), nitric oxide synthase (NOS), dimethylarginine dimethylaminohydrolase (DDAH), protein methyltransferases (PRMT), asymmetric dimethylarginine (ADMA), symmetric dimethylarginine (SDMA), dimethylamine (DMA), metabolic reprogramming, chemoprevention

## Abstract

**Simple Summary:**

Nitric oxide and arginine metabolism in colorectal cancer (CRC) holds potential for therapeutic intervention. We hypothesized that it can be modulated by oxicams, a class of non-steroidal anti-inflammatory drugs with documented chemopreventive and antineoplastic activity. The aim of this study was to determine the transcriptional patterns of pathway enzymes in CRC and evaluate the impact of classic and new oxicam analogues. Arginine metabolic pathways were altered not only in tumors but also in non-transformed mucosa from tumor vicinity, contributing to the phenomenon of tumor molecular margin. Classic oxicams, piroxicam and meloxicam, had negligible impact but their new analogues downregulated expression of dimethylarginine dimethylaminohydrolases and protein methyltransferases and upregulated asymmetric dimethylarginine. Those beneficial effects were accompanied by upregulation of arginase-2 and the potentially disadvantageous accumulation of arginine and symmetric dimethylarginine. Our findings provide novel insight into metabolic reprogramming in CRC and demonstrate that oxicam analogues are worth further consideration as novel anticancer agents.

**Abstract:**

L-arginine/nitric oxide pathway metabolites are altered in colorectal cancer (CRC). We evaluated underlying changes in pathway enzymes in 55 paired tumor/tumor-adjacent samples and 20 normal mucosa using quantitative-PCR and assessed the impact of classic and novel oxicam analogues on enzyme expression and intracellular metabolite concentration (LC-MS/MS) in Caco-2, HCT116, and HT-29 cells. Compared to normal mucosa, *ARG1*, *PRMT1,* and *PRMT5* were overexpressed in both tumor and tumor-adjacent tissue and *DDAH2* solely in tumor-adjacent tissue. Tumor-adjacent tissue had higher expression of *ARG1*, *DDAH1*, and *DDAH2* and lower *NOS2* than patients-matched tumors. The *ARG1* expression in tumors increased along with tumor grade and reflected lymph node involvement. Novel oxicam analogues with arylpiperazine moiety at the thiazine ring were more effective in downregulating *DDAHs* and *PRMTs* and upregulating *ARG2* than piroxicam and meloxicam. An analogue distinguished by propylene linker between thiazine’s and piperazine’s nitrogen atoms and containing two fluorine substituents was the strongest inhibitor of *DDAHs* and *PRMTs* expression, while an analogue containing propylene linker but no fluorine substituents was the strongest inhibitor of *ARG2* expression. Metabolic reprogramming in CRC includes overexpression of *DDAHs* and *PRMTs* in addition to *ARG1* and *NOS2* and is not restricted to tumor tissue but can be modulated by novel oxicam analogues.

## 1. Introduction

Colorectal cancer (CRC) remains one of the most common and lethal cancers, despite recent progress in the disease prevention and patients’ management [1]. Latest advances in various “-omics”, particularly genomics and proteomics, have improved our understanding of molecular landscape of CRC. Still, there are few resulting biomarkers to aid diagnosis and clinical-decision making or which serve as molecular targets for chemoprevention and treatment [2]. Metabolic reprogramming is one of eight recognized cancer hallmarks [3]. However, cancer metabolism is highly flexible and cancer type- and context-dependent [4]. Therefore, further research employing quantitative metabolic profiling is needed to translate metabolic abnormalities into clinical practice successfully [5].

**Figure 1:**
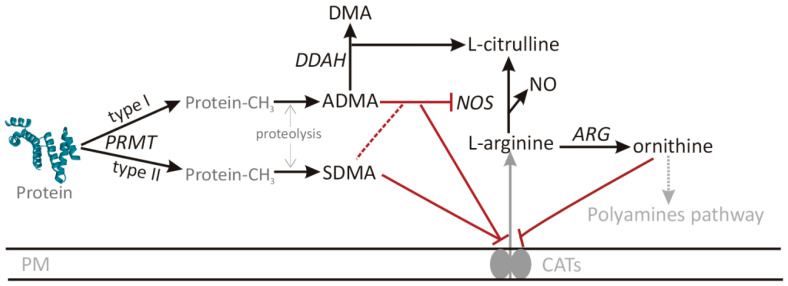
An overview of arginine/nitric oxide pathway. Metabolites are written in a straight script and enzymes in italics. Pathway components not determined in current study are marked in gray. Red color was used for inhibitory effects and a dashed line indicates weak impact. ADMA, asymmetric dimethylarginine; ARG, arginase; CATs, cationic amino acid transporter; DMA, dimethylamine; DDAH, dimethylarginine dimethylaminohydrolase; NOS, nitric oxide synthase; PM, plasma membrane; PRMT, protein methyltransferase.

We have recently demonstrated that CRC, as well as the disease-promoting conditions [6], is associated with systemic changes in metabolites of L-arginine/nitric oxide (NO) pathway, potentially applicable as CRC biomarkers and predictors of adverse clinical events after curative tumor resection [7]. In the present study, we aimed at transcriptional profiling of the underlying alterations in pathway enzymes as a follow-up. L-arginine (arginine) is a semi-essential amino acid, from which several biologically active substances are obtained. The main arginine-derived products are NO, co-synthesized with citrulline by a family of NO synthases (NOSs), and ornithine, co-synthesized with urea by arginases (ARGs) and subsequently used for polyamine synthesis. The NOS enzymes are inhibited, to varying degree, by asymmetric (ADMA) and symmetric (SDMA) dimethylarginines. Additionally, both metabolites compete with arginine for its membrane transporters. Dimethylamines are degradation products of proteins, methylated by protein methyltransferases (PRMTs). Type I enzymes (e.g., PRMT1) release ADMA and type II (e.g., PRMT5) release SDMA. The SDMA is removed by renal excretion while ADMA is degraded into dimethylamine (DMA) and citrulline by dimethylarginine dimethylaminohydrolases (DDAHs) [8,9]. An overview of L-arginine/NO pathway encompassing enzymes and metabolites determined in the present study is depicted in Figure 1.

The L-arginine pathway is potentially relevant for CRC development and progression [10,11,12]. However, arginine seems to play a dual role in carcinogenesis as its depletion hampers anticancer immunity [13]. Consequently, antineoplastic therapies based on arginine-deprivation or, on the contrary, the amino acid supplementation, are equally intensively investigated [8,13,14]. Moreover, there is also a growing interest in manipulating arginine metabolism as a chemoprevention, using specific inhibitors in combination with non-steroidal anti-inflammatory drugs (NSAIDs) [15,16,17]. While NSAIDs are proven anti-inflammatory agents, not all of their anticancer activity might be attributed to the downregulation of cyclooxygenases-mediated prostaglandin synthesis and the exact mechanisms involved in cancer chemoprevention remain elusive [18,19]. Recently, the chemopreventive activity of NSAIDs has been attributed, at least in part, to their interference with polyamine metabolism (reviewed in [19]). Here, we explored their potential effect on upstream L-arginine/NO pathway.

Oxicams are a class of NSAIDs, distinguished by the lack of a carboxyl group, which own their slightly acidic nature to an enolized β-dicarbonyl moiety. Structurally, they are benzothiazine-3-carboxylic acid amides (e.g., piroxicam, meloxicam) or thienothiazine-3-carboxylic acid amides [20]. Piroxicam and meloxicam have been shown to inhibit cancer cell growth [21,22,23,24,25] and angiogenesis [26] and to prevent surgery-induced emergence of secondary tumors [27], but their effect on the L-arginine/NO/ornithine pathway is largely unknown.

**Figure 2:**
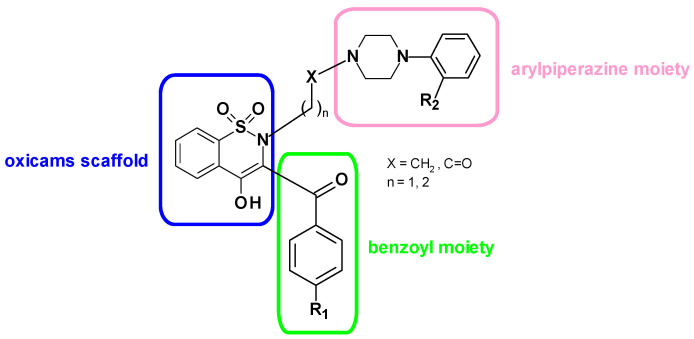
General structure of new oxicam analogues.

Therefore, this study was designed to determine metabolic reprogramming regarding L-arginine pathway enzymes in the colonic mucosa of CRC patients (*ARG1*, *NOS2*, *DDAH1*, *DDAH2*, *PRMT1*, and *PRMT5*) and to investigate the possible effect of selected oxicams on the pathway status in colorectal adenocarcinoma cell lines HCT 116, HT-29 and Caco-2, at the level of both metabolome (arginine, citrulline, ornithine, ADMA, SDMA, DMA, nitrates, and nitrites) and transcriptome (*ARG2*, *NOS2*, *DDAH1*, *DDAH2*, *PRMT1*, and *PRMT5*). In addition to classic drugs—meloxicam and piroxicam—five recently synthesized [28] oxicam analogues have been evaluated. Novel drugs, denoted as compounds #1–5 (Figure S1), possess a 1,2-benzothiazine oxicams scaffold and modified substituents in the 2- and 3-positions of the thiazine ring (Figure 2).

## 2. Results

### 2.1. Arginine/No Pathway Enzymes in CRC Patients

A transcriptional analysis of 55 pairs of patients’-matched colorectal samples—tumor and macroscopically normal mucosa adjacent to tumor—was conducted. Using reversely-transcribed quantitative (real-time) PCR and EvaGreen chemistry, the relative expression of *ARG1*, *DDAH1*, *DDAH2*, *NOS2*, *PRMT1*, and *PRMT5*, encoding key pathway enzymes, was determined.

#### 2.1.1. Pathway Enzymes in Patient-Matched Tumors and Non-Cancerous Tumor-Adjacent Tissue

Pairwise analysis showed significantly downregulated expression of *ARG1* (by 2.2-fold, *p* = 0.025), *DDAH1* (by 1.4-fold, *p* = 0.016), and *DDAH2* (by 1.6-fold, *p* = 0.005) and upregulated expression of *NOS2* (by 2.6-fold, *p* = 0.003) in tumor as compared to adjacent, macroscopically normal tissue. The expression of *PRMT5* tended to be upregulated as well (by 1.2-fold, *p* = 0.081) while that of *PRMT1* showed no difference (*p* = 0.850) (Figure S2).

#### 2.1.2. Association of Enzyme Expression Level with Cancer Pathology

Fold-change in expression (tumor-to-adjacent) was calculated and referred to cancer pathology. Fold-change in *ARG1* increased along with tumor grade (*ρ* = 0.36, *p* = 0.031) and growing N stage (N0-N1-N2): *ρ* = 0.35, *p* = 0.031. It was higher in patients with lymph node involvement (N1 and N2) than without (N0) by 7.1-fold (*p* = 0.017) and in stage III/IV than 0/I/II by 7.9-fold (*p* = 0.014). The *DDAH2* expression tended to be less decreased in N1/N2 than N0 cancers (by 1.5-fold, *p* = 0.059) and stage III/IV than 0/I/II cancers (by 1.5-fold, *p* = 0.058) (Figure S3).

#### 2.1.3. The Pathway Enzyme Expression in Colonic Mucosa from CRC Patients and Normal Colonic Tissue

As the molecular landscape is frequently altered also in macroscopically normal tissue adjacent to tumor [29,30], we compared the expression level of pathway enzymes in tumors and tumor-adjacent non-cancerous tissue with that in normal colonic tissue obtained from patients undergoing polypectomy (*n* = 20). 

**Figure 3:**
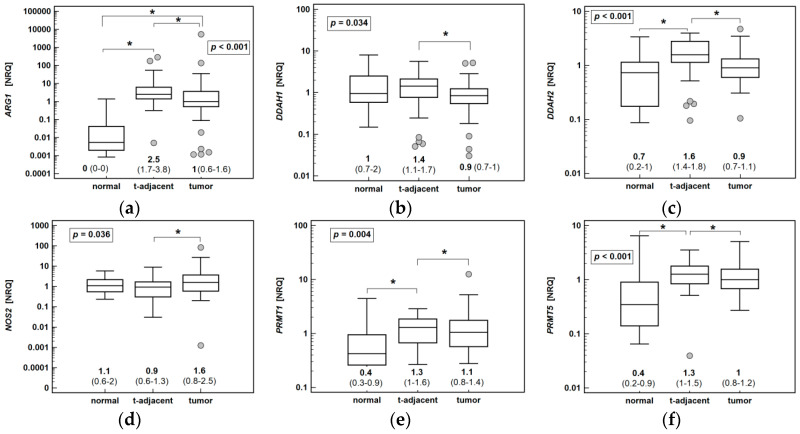
Comparison of arginine/nitric oxide pathway enzymes’ expression in normal colonic tissue and tumor and non-cancerous tumor-adjacent tissue: (**a**) *ARG1*; (**b**) *DDAH1*; (**c**) *DDAH2*; (**d**) *NOS2*; (**e**) *PRMT1*; (**f**) *PRMT2*. Data analyzed using a Kruskal–Wallis *H* test. Numeric data present medians of normalized relative quantities (NRQ), accompanied by 95% confidence interval (CI). Boxes indicate median with interquartile range; whiskers—95% CI; grey dots ‒ outlying observations. *, significant between-group differences (*p* < 0.05); *ARG2*, arginase 2; *DDAH*, dimethylarginine dimethylaminohydrolase; *NOS2*, inducible nitric oxide synthase; *PRMT*, protein methyltransferase.

The expression of *ARG1*, *PRMT1* and *PRMT5* was significantly elevated in both cancerous and non-cancerous tissue from CRC patients as compared to normal colonic mucosa. The expression of *DDAH2* was significantly elevated, and that of *DDAH1* non-significantly higher, exclusively in non-cancerous tumor-adjacent tissue (Figure 3).

### 2.2. Impact of Classic and Novel Oxicam Analogues on Arginine/NO Pathway

To answer the question whether oxicam drugs can affect the status of arginine/NO pathway, Caco-2, HCT 116 and HT-29 cells were stimulated for 24 h with 5, 50 and 200 µM concentration of classic (piroxicam and meloxicam) and novel oxicam drugs (compounds #1–5). Drug concentration range was selected based on literature data [21,24,31] and the results of sulforhodamine B (SRB) viability assay conducted in preliminary experiments (Figures S4–S6). In case of 5 µM and 200 µM concentration, an additional stimulation for 72 or 6 h, respectively, was conducted.

#### 2.2.1. Arginine/NO Pathway Status in Colonic Adenocarcinoma Cell Lines

**Figure 4:**
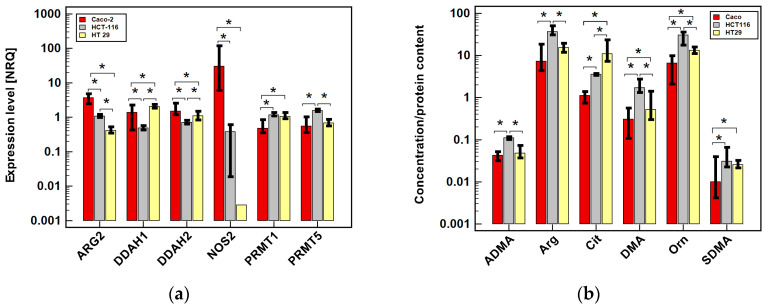
Comparison of arginine/nitric oxide pathway status in untreated colonic adenocarcinoma cell lines in 24-h cultures: (**a**) gene expression normalized to *GAPDH*; (**b**) intercellular metabolite concentration adjusted to protein content. Bars represent medians of normalized relative quantities (NRQ) with 95% confidence interval (whiskers). Data were analyzed using Kruskal–Wallis *H* test. *, significant between-group differences (*p* < 0.05); *ARG2*, arginase 2; *DDAH*, dimethylarginine dimethylaminohydrolase; *NOS2*, inducible nitric oxide synthase; *PRMT*, protein methyltransferase; ADMA, asymmetric dimethylarginine; Arg, arginine; Cit, citrulline; DMA, dimethylamine; Orn, ornithine; SDMA, symmetric dimethylamine.

First, we examined the expression level of pathway enzymes and the intracellular concentration of its metabolites in untreated cells (Figure 4).

The Caco-2 line had the highest expression of *ARG2* and *DDAH2* and was the only line expressing *NOS2* at quantifiable level. In turn, it had the lowest *PRMTs* levels. The HT-29 line had the lowest *ARG2* and *NOS2* expression and the highest *DDAH1*. The HCT 116 cells had the lowest *ARG2* and *DDAHs* and the highest *PRMT5* expression (Figure 4a).

Of the investigated cell lines, Caco-2 had generally the lowest concentration of pathway metabolites and HCT 116 the highest. The exception was citrulline—higher in HT-29 than HCT 116; arginine—the lowest in HT-29; and ADMA—present at comparable level in both Caco-2 and HT-29 cells (Figure 4b).

#### 2.2.2. Effect of Oxicam Drugs on Gene Expression of Key Pathway Enzymes

**Figure 5:**
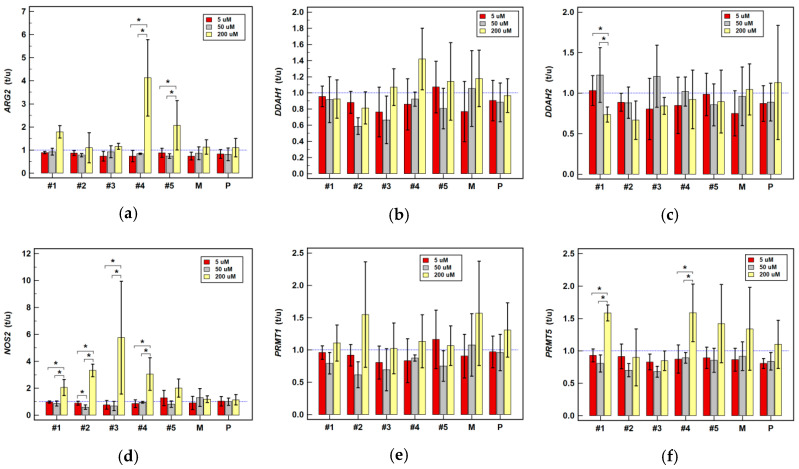
Dose-dependent effect of oxicams on enzyme gene expression in Caco-2 cells stimulated with 5, 50 and 200 µM drug concentrations for 24 h: (**a**) Arginase-2 (*ARG2*); (**b**) Dimethylarginine dimethylaminohydrolase-1 (*DDAH1*); (**c**) Dimethylarginine dimethylaminohydrolase-2 (*DDAH2*); (**d**) Nitric oxide synthase-2 (*NOS2*); (**e**) Protein methyltransferase-1 (*PRMT1*); (**f**) Protein methyltransferase-5 (*PRMT5*). Bars represent mean (*n* = 3) concentration ratios treated-to-untreated cells (t/u) with standard deviation (whiskers). No effect is marked by horizontal blue reference line. Data were analyzed using one-way analysis of variance. *, significant between-group differences; #1–5, oxicam analogues; M, meloxicam; P, piroxicam.

**Figure 6:**
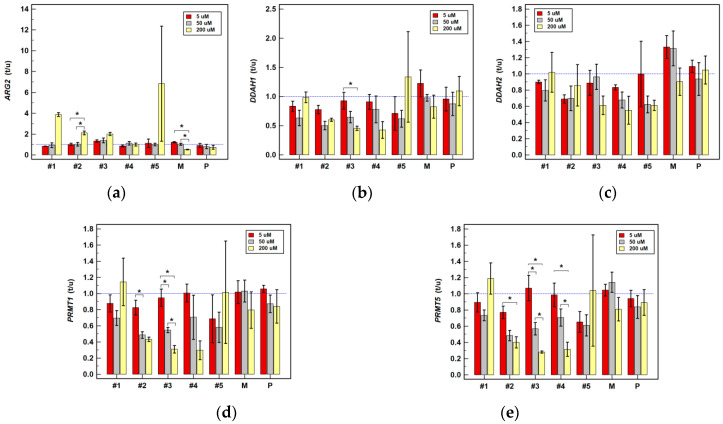
Dose-dependent effect of oxicams on enzyme gene expression in HCT 116 cells stimulated with 5, 50 and 200 µM drug concentrations for 24 h: (**a**) Arginase-2 (*ARG2*); (**b**) Dimethylarginine dimethylaminohydrolase-1 (*DDAH1*); (**c**) Dimethylarginine dimethylaminohydrolase-2 (*DDAH2*); (**d**) Protein methyltransferase-1 (*PRMT1*); (**e**) Protein methyltransferase-5 (*PRMT5*). Bars represent mean (*n* = 3) concentration ratios treated-to-untreated cells (t/u) with standard deviation (whiskers). No effect is marked by horizontal blue reference line. Data were analyzed using one-way analysis of variance. *, significant between-group differences; #1–5, oxicam analogues; M, meloxicam; P, piroxicam.

**Figure 7:**
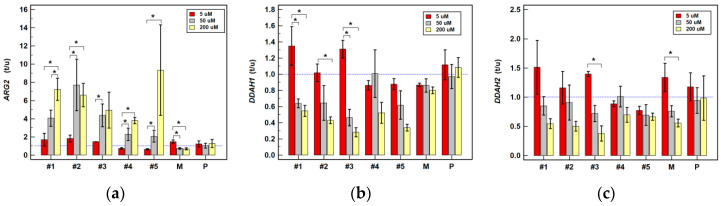

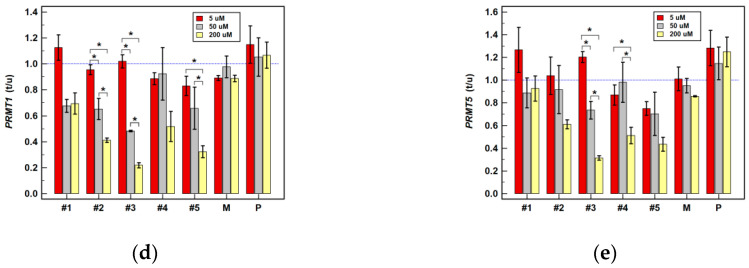
Dose-dependent effect of oxicams on enzyme gene expression in HT-29 cells stimulated with 5, 50 and 200 µM drug concentrations for 24 h: (**a**) Arginase-2 (*ARG2*); (**b**) Dimethylarginine dimethylaminohydrolase-1 (*DDAH1*); (**c**) Dimethylarginine dimethylaminohydrolase-2 (*DDAH2*); (**d**) Protein methyltransferase-1 (*PRMT1*); (**e**) Protein methyltransferase-5 (*PRMT5*). Bars represent mean (*n* = 3) concentration ratios treated-to-untreated cells (t/u) with standard deviation (whiskers). No effect is marked by horizontal blue reference line. Data were analyzed using one-way analysis of variance. * significant between-group differences; #1–5, oxicam analogues; M, meloxicam; P, piroxicam.

Pairwise analysis of treated and untreated cells showed piroxicam to have no significant effect on the expression of any examined genes (Table 1). Comparison of expression ratios (treated-to-untreated) at various drug concentrations showed no significant differences as well (Figure 5, Figure 6 and Figure 7).

Meloxicam slightly downregulated the expression of *DDAHs* and *PRMTs* in HT-29. It also downregulated *ARG2* in HCT 116 by two-fold at 200 µM concentration while slightly upregulating it at 5 µM (Table 1). The expression ratios in Caco-2 tended to increase along with increasing meloxicam concentration for the majority of investigated genes (Figure 5) but to decrease in HCT 116 (Figure 6) and HT-29 cells (Figure 7). Significant differences in expression ratios were observed regarding *ARG2* in HCT 116 and HT-29 cells and regarding *DDAH2* in HT-29 cells, as they were lower at higher drug concentrations. 

The examined cell lines were more responsive to the novel oxicam analogues, particularly HCT 116 and HT-29 cells (Table 1). 

The expression of *ARG2* tended to be slightly downregulated at lower and substantially upregulated at higher drug concentration. Compound #1 at 200 µM was its strongest inducer (Table 1). The *ARG2* expression ratios increased along with increasing compounds’ #1–5 concentration, significantly so in HT-29 (Figure 7) and for compound #2 in HCT 116 (Figure 6), and compounds #4 and #5 in Caco-2 (Figure 5). 

Compounds #1–3 and #5 had negative impact on *DDAHs* expression, mainly in HT-29 cells and at 200 µM concentration (Table 1). The *DDAH1* expression ratio decreased along with increasing drug concentration, significantly so for compounds #1–3 in HT-29 cells (Figure 7) and for compound #3 in HCT 116 (Figure 6). The dose-dependent effect on *DDAH2* expression in HT-29 cells was notable for compounds #1–3, but significant solely for compound #3 (Figure 7).

The *NOS2* expression was quantifiable only in Caco-2 cells and was significantly upregulated by compounds #1, #2 and #4 at 200 µM concentration (Table 1). Dose-dependent effect was notable for compounds #1–4 (Figure 5).

At 200 µM, compounds #2–5 downregulated expression of *PRMTs* in HCT 116 and HT-29 cells and compounds #2–3 also at 50 µM concentration (Table 1). The *PRMT1* expression ratios decreased along with increasing drug concentration, significantly so for compounds #2 and #3 in HCT 116 (Figure 6) and #2, #3, and #5 in HT-29 cells (Figure 7). Likewise, the *PRMT5* expression ratios decreased in a dose dependent-manner, significantly so for compounds #2–4 in HCT 116 (Figure 6) and for compounds #3 and #4 in HT-29 (Figure 7).

In addition, the effect of oxicams on cancer cells during longer stimulation with low drug concentration (72-h, 5 µM) or during shorter stimulation with high drug concentration (6-h, 200 µM) was evaluated. Prolonged stimulation with low meloxicam concentration slightly decreased, or tended to decrease, *ARG2* and *DDAH2* expression in HCT 116 and HT-29 cells and *PRMT5* in HCT 116 cells. Compound #4 tended to downregulate *ARG2* and *PRMT5*. Shorter stimulation with high drug concentration upregulated *ARG2* expression in HCT 116 (compound #1 by 1.6-fold) and HT-29 cells (compound #3 by 3.5-fold and compound #5 by 2.6-fold while compounds #1 and #2 displayed a similar tendency) (Table S1).

#### 2.2.3. Effect of Oxicam Drugs on Enzyme Protein Expression in HT-29 Cells

As the examined oxicam drugs had the most pronounced effect on HT-29 cells, this line was chosen to seek confirmation of drug effect on protein level. Western-blot analysis showed that at 200 µM, compounds #1, #2, #4 and #5 upregulated arginase-2 protein expression as compared to untreated HT-29 cells (Figure S7). When normalized to total protein, allowing for adjustment against differences in gel loading and transfer efficiency, they upregulated arginase-2 concentration by 128, 1.2, 11, and 17.1-fold. Piroxicam upregulated and meloxicam downregulated arginase-2 by 2.7 and 12.7-fold, respectively. Except for compound #5, upregulating arginase-2 by 2.7-fold, 5 µM drug concentrations had negative impact on the enzyme expression. Compound #1 downregulated arginase-2 protein expression by 5.9-fold, compounds #2 and #3 by 7.7-fold, and piroxicam and meloxicam by 1.5 and 9.4-fold, respectively.

Compounds #1–5 downregulated DDAH1 and DDAH2 protein expression at 200 µM (Figure S7) by 2.2, 22.8, 72.8, 4.4, and 299-fold (DDAH1) and by 10.8, 29.5, 8.9, 2.6, and 24.3-fold (DDAH2), respectively. Piroxicam downregulated DDAH1 protein by 1.9-fold and DDAH2 by 3.6-fold. Meloxicam downregulated DDAH2 as well (by 1.9-fold). 

Likewise, oxicam drugs at 200 µM concentration downregulated PRMTs (Figure S7). Compounds #1–3 and piroxicam were more effective in case of PRMT1 (downregulation by 10.8, 29.5, 8.9, and 3.6-fold for PRMT1 and 2.4, 1.7, 1.4, and 1.9-fold for PRMT5, respectively) and compounds #4 and #5 in case of PRMT5 (downregulation by 2.6 and 24.3-fold for PRMT1 and by 19.7 and 50-fold for PRMT5, respectively). Meloxicam had no substantial effect on PRMT1 and upregulated PRMT5 by 1.8-fold.

#### 2.2.4. Effect of Oxicam Drugs on Intracellular Concentrations of Key Pathway Metabolites

Pairwise analysis of treated as compared to non-treated cells showed mostly positive drug effect on metabolite accumulation, especially at 50 and 200 µM concentration (Table 2).

**Figure 8:**
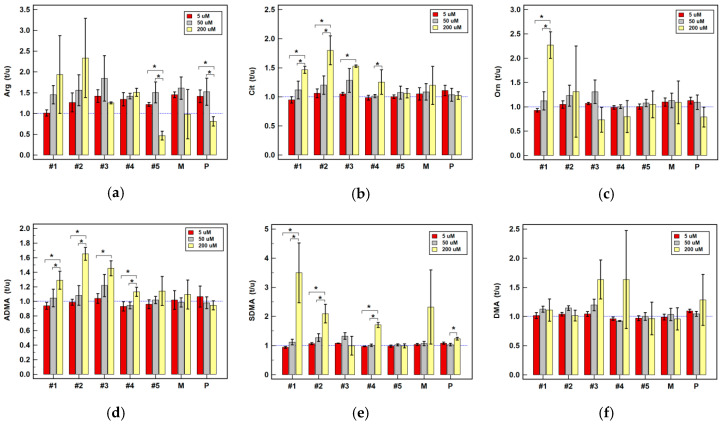
Dose-dependent effect of oxicams on the intracellular metabolite concentration in Caco-2 cells stimulated with 5, 50 and 200 µM drug concentrations for 24 h: (**a**) Arginine (Arg); (**b**) Citrulline (Cit); (**c**) Ornithine (Orn); (**d**) Asymmetric dimethylarginine (ADMA); (**e**) Symmetric dimethylarginine (SDMA); (**f**) Dimethylamine (DMA). Bars represent mean (*n* = 3) concentration ratios treated-to-untreated cells (t/u) with standard deviation (whiskers). No effect is marked by horizontal blue reference line. Data were analyzed using one-way analysis of variance. * significant between-group differences; #1–5, oxicam analogues; M, meloxicam; P, piroxicam.

**Figure 9:**
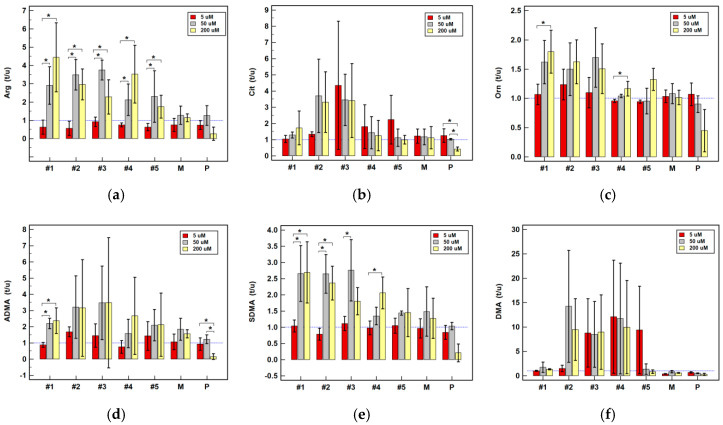
Dose-dependent effect of oxicams on the intracellular metabolite concentration in HT-29 cells stimulated with 5, 50 and 200 µM drug concentrations for 24 h: (**a**) Arginine (Arg); (**b**) Citrulline (Cit); (**c**) Ornithine (Orn); (**d**) Asymmetric dimethylarginine (ADMA); (**e**) Symmetric dimethylarginine (SDMA); (**f**) Dimethylamine (DMA). Bars represent mean (*n* = 3) concentration ratios treated-to-untreated cells (t/u) with standard deviation (whiskers). No effect is marked by horizontal blue reference line. Data were analyzed using one-way analysis of variance. * significant between-group differences; #1–5, oxicam analogues; M, meloxicam; P, piroxicam.

Piroxicam caused minor accumulation of arginine at 5 µM concentration and upregulation of SDMA at 200 µM concentration in Caco-2 cells, while downregulating DMA by 2.1-fold in HCT 116 at 50 µM concentration (Table 2). The expression ratio of arginine at 200 µM drug concentration was significantly lower in Caco-2 cells and that of SDMA was higher (Figure 8). The expression ratios of ADMA and citrulline in HT-29 cells were lower as well (Figure 9).

**Figure 10:**
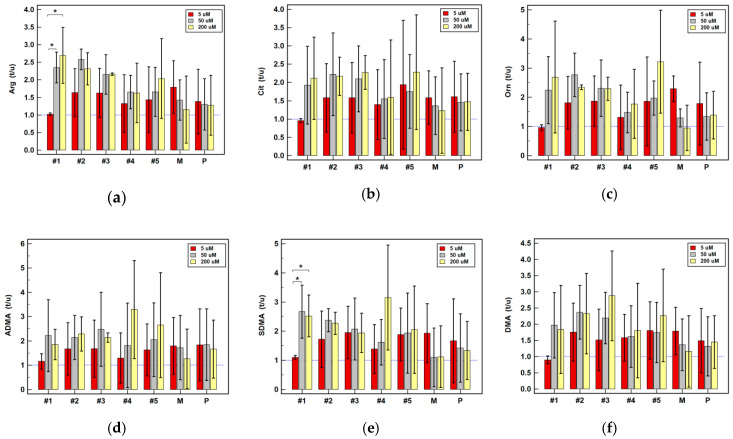
Dose-dependent effect of oxicams on the intracellular metabolite concentration in HCT 116 cells stimulated with 5, 50 and 200 µM drug concentrations for 24 h: (**a**) Arginine (Arg); (**b**) Citrulline (Cit); (**c**) Ornithine (Orn); (**d**) Asymmetric dimethylarginine (ADMA); (**e**) Symmetric dimethylarginine (SDMA); (**f**) Dimethylamine (DMA). Bars represent mean (*n* = 3) concentration ratios treated-to-untreated cells (t/u) with standard deviation (whiskers). No effect is marked by horizontal blue reference line. Data were analyzed using one-way analysis of variance. *, significant between-group differences; #1–5, oxicam analogues; M, meloxicam; P, piroxicam.

Meloxicam, at 5 and 50 µM concentration, slightly upregulated intracellular arginine level. At 5 µM concentration, it contributed to 2.3-fold accumulation of ornithine in HCT 116 while at 200 µM concentration, it upregulated ADMA by 1.7-fold. The DMA in HT-29 was downregulated by meloxicam at 5 µM concentration and similar tendency could be observed at 200 µM of drug concentration (Table 2). However, no significant dose-dependent effect could be observed (Figure 8, Figure 9 and Figure 10).

Non-classic oxicams had a more pronounced effect on intracellular metabolite accumulation (Table 2). Arginine was upregulated, to varying degree, by compounds #1–4 at 50 and 200 µM concentration in all cell lines, albeit their impact was more pronounced in HT-29 (Table 2). The metabolite accumulation increased dose-dependently, significantly so for compound #1 in HCT 116 (Figure 10) and #1–5 in HT-29 cells (Figure 9). In the case of compounds #2, #3 and #5, the maximal stimulatory effect was at 50 µM concentration. Noteworthy, the expression ratios at 5 µM were indicative of an inhibitory effect on arginine accumulation in HT-29 (Figure 9). Unlike other tested oxicams, 200 µM compound #5 in Caco-2 cells downregulated arginine (Table 2, Figure 8).

Intracellular citrulline concentration increased along with increasing drug concentration in Caco-2 cells, significantly so for compounds #2–4 (Figure 8). In pairwise analysis, significant citrulline upregulation was observed in Caco-2 cells for 200 µM compounds #1-3 and in HCT 116 cells for 200 µM compounds #2 and #3 (Table 2).

Ornithine was upregulated by compounds #1–3 and #5 at 50 and 200 µM concentrations in HCT 116 and HT-29 cells (Table 2), while only 200 µM compound #1 was effective in Caco-2 cells (Table 2), increasing the amino acid concentration in a dose-dependent manner (Figure 8). A significant increase in ornithine accumulation ratio along with increasing drug concentration could be observed also for compounds #1 and #4 in HT-29 cells (Figure 9). 

Intracellular ADMA was upregulated by 200 µM compounds #1-3 in Caco-2 and HCT 116 cells and by 50 µM in HT-29 cells (Table 2). The accumulation rates in stimulated Caco-2 cells increased dose-dependently for compounds #1–4 (Figure 8) and in HT-29 cells for compound #1 (Figure 9). 

Likewise, SDMA concentration increased in response to stimulation and the effect was more pronounced than in case of ADMA. It was upregulated by 50 µM compounds #1–3 and #5 in HCT 116 and HT-29 cells and by 200 µM compounds #1–4 in all cell lines (Table 2). Accumulation ratios increased along with drug concentration, significantly so for compound #1 in Caco-2 (Figure 8) and #1 and #4 in HT-29 cells (Figure 9).

Pairwise analysis showed that compounds #2 and #3 in HT-29 cells tended to increase intracellular DMA level (Table 2). However, there was no significant dose-depended effect as there was a great variability in DMA concentration between experiments (Figure 8, Figure 9 and Figure 10).

In addition, the effect of oxicams at 200 µM was analyzed following shorter (6-h) incubation. Compound #1 increased arginine by 1.8-fold, ADMA by 1.6-fold, and SDMA by 1.8-fold in HT-29 cells. Longer (72-h) stimulation with low (5 µM) concentration, analyzed for compounds #4–5 and classic oxicams had no significant effect in any investigated cell line (Table S2).

#### 2.2.5. Effect of Oxicam Drugs on Nitrite and Nitrate Concentrations in Conditioned Media

Gaseous NO has a very short half-life and its more stable metabolites, nitrite (NO_2_^-^) and nitrate (NO_3_^-^), determined colorimetrically after Griess reaction, are used as an indirect measure of NO production [32]. Total nitric oxide (NO_2_^-^/NO_3_^-^) was calculated but, with the exception of slightly decreased NO by compound #1 at 200 µM after 6-h stimulation of HCT 116 cells and a similar tendency observed in Caco-2 for compounds #1 and #2 at 5 and 50 µM after 24-h stimulation, no changes were observed. 

## 3. Discussion

Cancer metabolic reprogramming is believed to hold a great potential as a source of novel biomarkers and molecular targets for chemoprevention and treatment [5]. Altered concentration of L-arginine/NO pathway metabolites at systemic level and their utility as diagnostic and prognostic tools in CRC has already been demonstrated [7]. An accumulation of arginine, citrulline, ADMA, and SDMA in colorectal cancer tissue has been shown as well [10,33,34]. Here, we intended to unravel the molecular background of these alterations, aiming at a comprehensive analysis going beyond the well-studied NO synthases and arginases, and to evaluate the ability of classic and novel oxicam NSAIDs to modulate the arginine/NO pathway. 

As expected, pairwise analysis of matched cancerous and non-cancerous tissue showed significant elevation of *NOS2* transcripts in tumors, corroborating earlier reports (reviewed in [35]). The NO generated by NOS2 isoform contributes to the formation of peroxinitrite, assisting in macromolecule damage and genome destabilization and thus facilitating neoplastic transformation [36]. Accordingly, local expression of *NOS2* is upregulated in inflammatory bowel conditions [6] and linked with increased risk of CRC development [35]. The NO is also implicated in the promotion of angiogenesis and therefore in aiding cancer cell dissemination [37]. In this respect, targeting NOS2 may be considered beneficial in both chemopreventive and antiangiogenic capacity. In animal models of colon carcinogenesis, piroxicam has been shown to downregulate protein iNOS expression and reduce NO synthesis, determined indirectly by nitrite and nitrate production [38]. Here, solely Caco-2 cells had quantifiable *NOS2* expression, which—however—was affected neither by piroxicam nor meloxicam. In turn, novel oxicam drugs stimulated *NOS2* expression at a 200 µM concentration. However, upregulated *NOS2* expression did not translate into accelerated NO synthesis as total NO tended to be reduced following Caco-2 stimulation, probably owing to the concomitant ADMA accumulation. Noteworthy, the dose-dependent responses of Caco-2 cells to oxicam stimulation were frequently opposite to those of HT-29 or HCT 116 and therefore the observation made on Caco-2 should not be easily generalized.

The ADMA is a natural inhibitor of NO synthases but its role in cancer is severely understudied and equivocal. On one hand, it has been shown to confer protection to colonic cancer cells against nutritional stress and doxorubicin-induced death [39], giving rationale for observed metabolite accumulation in colorectal tumors [33,34]. On the other hand, it interferes with vascular endothelial growth factor (VEGF)-induced angiogenesis. Consequently, targeting DDAH1, its key degrading enzyme, is viewed as an antiangiogenic strategy (reviewed in [37]). However, our analysis of clinical samples showed the downregulation of *DDAH1* and *DDAH2* in colonic tumors. While consistent with ADMA accumulation [33,34], our observation contradicts the results of RNA-seq expression data analysis, indicating higher *DDAH1* expression level in tumors than normal tissue in colonic and rectal adenocarcinomas [37]. Still, in line with the cancer-promoting activities attributed to DDAH1 [40,41,42,43] and DDAH2 [44], the expression ratio (tumor-to-adjacent) of *DDAH2* tended to increase with cancer advancement, reflecting lymph node involvement. The above-mentioned RNA-seq expression data analysis has revealed cancer type-dependent up- or downregulation of *DDAHs* expression as well as a lack of consistency between transcriptional patterns of *DDAH1* and *DDAH2* [37]. Moreover, the data concerning DDAHs’ role in cancer are far from equivocal and might also be cancer type-dependent and context-related. As mentioned, DDAH1 is implicated in promoting angiogenesis via the indirect upregulation of NO and VEGF-A [40,45,46]. It has also been shown to affect the Wnt/β-catenin pathway and, as such, might be implicated in epithelial-mesenchymal transition (EMT) [47]. However, complicating DDAH1 relevance in cancer, the enzyme depletion and not its upregulation induced EMT in gastric cancer. Moreover, forced DDAH1 overexpression inhibited cell migration and caused degradation of β-catenin [48]. Furthermore, corroborating our observations in CRC and also opposing the results of RNA-seq expression data analysis, Ye et al. [48] showed DDAH1 downregulation in patient-derived tumor samples, associated with more aggressive phenotype and poor patient survival. Contrary results have been reported for prostate [41] and breast cancers [42], in which DDAH1 was overexpressed in cell lines with aggressive phenotype and its knockout resulted in inhibition of cell migration, implicating the enzyme in breast and pancreatic cancer invasion and metastasis [42,43]. Furthermore, DDAH2 has been shown to promote angiogenesis via upregulating the expression of the endothelial isoform of NOS, as demonstrated in lung adenocarcinoma cell lines, and to be associated with aggressive lung cancer phenotype as well as poor patient survival [44]. 

Noteworthily, the ADMA and SDMA accumulation has been observed in both tumor and non-cancerous tumor-adjacent tissue [33]. Therefore, we compared the level of enzyme expression in tumors and adjacent tissue to that in normal colonic mucosa. Normal tissue was obtained from patients undergoing polypectomy of benign polyps. The rationale for comparative analysis was that macroscopically normal mucosa surrounding tumor might already be altered at the molecular level, creating a tumor-promoting environment [29,30]. Many cases of such changes have been documented in various cancers, including CRC [49,50,51]. Interestingly, we found that the expression level of *DDAHs* was comparable between normal mucosa and tumors, while both genes were upregulated in still non-transformed tumor-adjacent tissue. Supporting possible DDAHs involvement in predisposing to cancer development, colonic *DDAH1* expression has been upregulated in patients with Crohn’s disease, solely during the disease flare, while that of *DDAH2* was also upregulated during remission. In patients with ulcerative colitis, only *DDAH2* has been upregulated, and exclusively during the disease flare [6]. 

The *DDAH* downregulation in tumors, even if not apparent, could not explain reported co-accumulation of SDMA, not catabolized by these enzymes. The ADMA and SDMA pool is regulated by the rate of their catabolism/excretion as well as synthesis, in which a prominent role is played by the PRMTs. While there seem to be no significant differences in *PRMT1* and *PRMT5* expression between colorectal tumors and adjacent-tissue in pairwise analysis, both genes appeared to be upregulated in CRC patients as compared to normal mucosa. Overexpression of *PRMT1* and *PRMT5* in both cancerous and non-transformed tissue would explain ADMA and SDMA accumulation reported elsewhere [33,34]. Early death of knockout mice stresses the importance of PRMT1 and PRMT5 enzymes [52]. Nonetheless, little is known on their status and role in cancer. Recent findings reveal that the significance of PRMT enzymes goes far beyond regulation of NO synthesis, as they seem to play a key role in the global regulation of RNA splicing and translation [53]. They have been reportedly overexpressed in certain cancers [52] and, therefore, the anticancer potential of PRMT inhibitors is currently being investigated [54]. The PRMT1 enzyme depletion has been shown to induce cell cycle arrest and reduce cancer cell proliferation, while an overexpression of one of the PRMT1 variants has been shown to reduce apoptosis (reviewed in [55]). Previously, Mathioudaki et al. [56] evaluated the expression pattern of alternative splicing variants of *PRMT1*, demonstrating that colon carcinogenesis and CRC aggressiveness is associated with a specific *PRMT1* variant, denoted *v1*. The primer pair used here was not variant-specific and allowed for the amplification of *v1*-*v4* isoforms of PRMT1, thus reflecting rather global *PRMT1* expression, which may explain the lack of association with CRC pathological data. The PRMT5 activity has been linked with cell transformation and thus implicated in cancer initiation [57]. Correspondingly, the upregulation of *PRMT1* and *PRMT5* expression also accompanied bowel inflammation in patients with Crohn’s disease and ulcerative colitis [6]. In addition, *PRMT5* overexpression has been associated with poor prognosis in gastric, lung, and breast cancers [57]. Only recently, a novel function of PRMT5 has been discovered—the enzyme has been implicated in regulating Hsp90A function as its knockdown results in the degradation of Hsp90A client proteins and cell apoptosis. As Hsp90A is a known cancer-related chaperone, targeting PRMT5 may have additional anticancer benefit in lifting a protection exerted by Hsp90A over a number of oncoproteins [58].

Therefore, it is of potential clinical relevance that the oxicams tested here occurred to inhibit transcription of *PRMTs* as well as *DDAHs*. While piroxicam had no significant impact, a substantial and significant effect was observed for 200 µM meloxicam, which, however, solely inhibited DDAH2 and exclusively in HT-29 cells. Still, both classic oxicams downregulated DDAHs and PRMTs at protein level, although to lesser degree than novel derivatives. Many experimental, epidemiologic, and clinical studies have suggested that NSAIDs, including oxicams, are promising chemopreventive and anticancer agents [38,59,60,61]. As mentioned before, not all antitumor activity could be contributed to cyclooxygenase inhibition. Accordingly, the inhibition of JAK3 and STAT3 [38] and c-Myc [60] expression and activity has been implicated in the anti-neoplastic mechanism of piroxicam. However, the intensification of side effects, especially on the gastrointestinal tract, has become an obstacle to perusing piroxicam application in chemoprevention [62,63]. Therefore, Szczęśniak-Sięga et al. [28] obtained new oxicams analogues without gastrotoxicity as potential safer chemopreventive agents. The modifications at the 2-position of the thiazine ring included the replacement of a small methyl substituent with an extended arylpiperazine pharmacophore, because Hatnapure et al. [64] noted that the high electron donating ability of the piperazine moiety can be explicitly correlated to high anti-inflammatory activity of compounds. At the same time, changes in the 3-position of the thiazine involved the replacement of a 2 pyridocarbamoyl substituent with the benzoyl moiety, since it has been shown that such modification increases the analgesic effect [65]. The thiazine and piperazine nitrogen atoms were connected by two types of linker—propylene linker, present in many potent analgesic arylpiperazine derivative, and oxoethylene linker, because of the introduction of a carbonyl moiety to the alkyl linker aimed at intensifying analgesia in accordance with the pharmacophore model proposed by Dogruer et al. [66].

Here, we showed that oxicam analogues were more effective than piroxicam and meloxicam in modulating DDAHs and PRMTs. They inhibited both *DDAH1* and *DDAH2,* and, even more markedly, both *PRMT1* and *PRMT5* in HT-29 and HCT 116 cells. These novel derivatives differ from classic oxicams with the presence of arylpiperazine moiety, indicating its significance for affecting the transcription of pathway enzymes. The compound #3 seems to be the most effective and act consistently, in a dose-dependent manner. It inhibited *DDAH1* expression in HCT 116 and HT-29 and *DDAH2* expression in HT-29 and *PRMT1* and *PRMT5* in both these cell lines. A dose-dependent effect was also observed in the case of compound #2 on *PRMT1* expression in both HCT 116 and HT-29 and compounds #2 and #5 on *PRMT1* in HCT 116 and HT-29 or solely HT-29, respectively, or compound #4 on *PRMT5* in HCT 116 and HT-29 cells. Referring to differences in the compound efficiency of these in chemical structure seems to indicate that the presence of electron-withdrawing substituents (present in compounds #2–5 but not in #1) is beneficial for inhibiting enzyme transcription. Compound #3 is distinguished by the presence of two such substituents, both fluorine atoms and thus stronger in terms of electron-withdrawing compared to the fluorine and chlorine present in compound #5. In addition, compound #3 contains a three-carbon linker (propylene) between thiazine and piperazine nitrogens, instead of two-carbon linker with an additional carbonyl group (2-oxoethylene) present in compounds #4 and #5, implying its significance for the efficient inhibition of pathway enzyme transcription.

As pointed out by Faubert et al. [4], it becomes increasingly apparent that alterations in metabolic pathways fueling the growth of localized tumors are distinct from those facilitating metastasis and resistance to anticancer treatment. Concerning the L-arginine/NO pathway, studies on mice models of breast cancer have revealed that early metastatic disease is characterized by a shift towards arginase and polyamine synthesis [67]. Intriguingly, novel oxicam analogues, at 200 µM concentration, had an even more substantial but stimulatory effect on *ARG2* expression. In turn, a low compounds concentration tended to have an opposite effect. It was clearly visible at protein level, where 5 µM drug concentrations downregulated arginase-2. Among the tested analogues, compound #1 upregulated the enzyme expression at both mRNA and protein level more markedly than others. Piroxicam had no effect, while meloxicam displayed an inverse pattern to novel oxicam analogues. It slightly upregulated *ARG2* gene expression in HCT 116 and HT-29 cells at a low concentration but downregulated at high. Those observations were confirmed at the protein level in HT-29 cells, where 200 µM meloxicam indeed downregulated arginase-2. The enzyme expression in clinical samples was too low to be reliably quantified, although this isoform is thought to be expressed in extrahepatic organs, including intestine. Moreover, Wu at al. [68] analyzed the Cancer RNA-seq Nexus (CRN) public database and found *ARG2* to be upregulated in CRC tumors. Functional experiments have shown that silencing *ARG2* resulted in the attenuation of HT-29 cell proliferation, migration, and invasion and increased apoptosis. In turn, *ARG2* knockout increased the expression of TCRζ on co-cultured Jurkat cells [68], implying arginase 2 in promotion of tumor growth and immune evasion. Here, we evaluated the expression of *ARG1* and found it to be elevated in both tumors and tumor-adjacent tissue as compared to normal mucosa. Arginase-1 is considered a hepatic enzyme, expressed also in myeloid cells such as M2 polarized macrophages, dendritic cells, and myeloid-derived suppressor cells (MDSC) [69]. Upregulated arginase 1, leading to the depletion of extracellular arginine, is a hallmark of tumor immunosuppression and an immunomodulatory target in anti-cancer therapy [13,70,71]. In addition, ornithine produced by the enzyme is a key source of tumor-promoting polyamines in proliferating cells [15,71,72]. Consistently, arginase activity is reportedly elevated in CRC patients, both in tumor tissue [72] and at systemic level [73], more so in patients with liver metastases [73]. Corroborating our observations, a more recent study has shown arginase 1 upregulation at mRNA and protein level, which was more pronounced at stage III and IV and in tumors accompanied by lymph node metastases. Moreover, the enzyme overexpression was associated with adverse prognoses for overall and disease-free survival [74]. Others have shown that tumors, e.g., in ovarian cancer, can use small extracellular vesicles to deliver the enzyme, as a metabolic checkpoint molecule, to distant immune cells in order to induce immunosuppression [75]. In the present study, *ARG1* additionally correlated positively with tumor grade, indicative of its aggressiveness. Taking into account the role of arginases, their upregulation by novel oxicam analogues, observed here, is rather undesirable. However, a protective role of MDSC-derived arginase 1, favoring accumulation of IL-17A and leading to relief of colitis has recently been demonstrated by Ma et al. [76]. Moreover, other NSAIDs, e.g., diclofenac, have been shown to increase protein expression and the activity of arginase 1, which, however, translated into the inhibited growth of xenografted pancreatic tumors. The enzyme probably acted by diminishing arginine availability for NO synthesis and exerted additional anti-angiogenic effects [77]. 

While *ARG2* expression in colonic cell lines was clearly upregulated by novel oxicam analogues at 200 µM concentration, further confirmed at the protein level, so was the concentration of arginine. This observation is counterintuitive, as arginase metabolizes arginine to produce ornithine, further used for polyamine synthesis. In turn, this time consistently with upregulated *ARG2*, the intracellular concentration of ornithine was increased as well. Noteworthily, ornithine accumulation may also indicate a block on the level of ornithine decarboxylase (ODC). This possibility warrants further investigation because of the significance of polyamine for tumor growth and resistance to chemotherapy [11,12,15]. It is substantiated by the observation that several NSAIDs [78], including piroxicam [61], have been shown to inhibit the enzyme. Moreover, ODC expression is controlled by *c-Myc* gene [19] and piroxicam has been demonstrated to interfere with its signaling [60].

Arginine was accumulated also in Caco-2 cells, in which 200 µM novel oxicam analogues additionally induced expression of *NOS2*, another arginine-metabolizing enzyme. However, the stimulatory effect on arginine conversion to NO might be, at least to some degree, counteracted by concomitant elevation in ADMA. The ADMA would inhibit NOS enzymatic activity, preserving arginine. The notion is supported by the lack of elevation in nitrites and nitrates despite *NOS2* upregulation. 

It is possible that investigated oxicam analogues influence the expression of amino acid membrane transporters, leading to arginine accumulation, despite the upregulated expression of ARG2 and NOS2. The notion is supported by the accumulation of both dimethylarginines and citrulline, which is also counterintuitive. The evaluated oxicam analogues inhibited DDAHs, which would potentially lead to ADMA accumulation. However, the effect was observed primarily in HT-29 cells, while ADMA accumulation was observed rather in Caco-2 line and accompanied by an elevation in citrulline, a product of DDAHs. Moreover, the downregulation of PRMT expression would lead to diminished ADMA and SDMA generation, unless they were prevented from being released from the cell. It is worth mentioning, that although the effects of SDMA are mostly unknown, its accumulation in colorectal tumors has been linked with increased risk of metastasis [33] and therefore is not beneficial. There are many arginine transporters, several of which mediate both influx and efflux [79] and can transport arginine as well as its methylated derivatives. Importantly, their expression might be upregulated by classic pro-inflammatory cytokines such as IL-1β and TNFα, with CAT-1 and CAT-2B being an example [79]. Therefore, oxicam analogues, by virtue of being anti-inflammatory agents, may indirectly decrease transporter expression, leading to diminished metabolite efflux.

## 4. Materials and Methods 

### 4.1. Patients

Paired bowel samples, including tumor and tumor-adjacent tissues (macroscopically normal), were harvested from 55 patients undergoing curative resection of histologically confirmed colorectal adenocarcinoma in the Regional Specialist Hospital in Wroclaw in Department of Oncological Surgery, between 2013 and 2015. Preoperative workup consisted of bloodwork, colonoscopy, and computed tomography (abdominal and pelvic) and—for rectal cancer—pelvic magnetic resonance imaging. The 7th edition of TNM cancer grading system was used for pathological staging. Patients’ characteristics are given in Table 3.

Harvested samples were washed with phosphate buffered saline (PBS) and immersed in RNAlater solution (Ambion Inc., Austin, TX, USA). Samples were kept at −80 °C until RNA isolation.

For comparative purposes, we retrieved previously collected data [6] on enzyme expression in normal large bowel mucosa from patients submitted for polypectomy of benign polyps (*n* = 20).

### 4.2. Ethical Approval

Sample collection for the study purpose was approved by the Medical Ethics Committees of Regional Specialist Hospital (#KB/nr 1/rok 2012 from 26 June 2012).

### 4.3. Cell Cultures

Human colon cancer cell lines: HT-29 (ATCC^®^ HTB-38™), HCT 116 (ATCC^®^ CCL-247™), and Caco-2 (ATCC^®^ HTB-37™) were obtained from ATCC (MD, USA). Cells were cultured in CELCULTURE^®^ CCL-170B-8 incubator (Esco, Singapore) at 37 °C in 95% air with 5% CO_2_ in 75 cm^2^ cell culture flasks (Thermo Fisher Scientific, Waltham, MA, USA). Dulbecco’s modified Eagle medium (Gibco, Thermo Fisher Scientific) with 10% fetal bovine serum (Gibco) and 1% GlutaMAX™-I (Gibco) was used with addition of stabilized 1% antibiotic antimycotic solution containing 10,000 units of penicilin/mL, 10 mg/mL of streptomycin, 25µg/mL of amphotericin B (Sigma-Aldrich, St. Luis, MO, USA). Medium was replaced with fresh one every third day. Upon growth termination, cells were washed with Dulbecco’s phosphate buffered saline (Gibco) and harvested using TrypLE™ Express (Gibco). Collected cells were stained with 0.4% trypan blue solution (Invitrogen, Thermo Fisher Scientific) and quantified using Countess™ Automated Cell Counter (Invitrogen).

Cells were seeded at 2 × 10^5^ cells/well on a 6-well plate for transcriptomic, metabolomic, and protein analysis. For specified times (6, 24, 72, and/or 48 h), cells were treated with piroxicam (Sigma-Aldrich), meloxicam (Alfa Aesar, Thermo Fisher Scientific), or novel analogues of oxicam dissolved in dimethyl sulfoxide (DMSO) at 5, 50, or 200 µM concentration. Cells treated with solvent alone (DMSO, up-to 0.25%) were used as controls and referred to as “untreated cells”. Compound #1 at 200 µM was dissolved in 1% DMSO due to its lower solubility. Cells treated with 1% DMSO were used as respective control.

For transcriptomic analysis, after the 6, 24 and 72 h media were removed and cells were lyzed using Trizol (Thermo-Fisher Scientific). Collected lysates were stored at −80 °C.

For metabolomic analysis, after the 6, 24 and 72 h media were removed and cells were washed twice with PBS and subjected to methanol extraction (cold methanol:water (3:1), repeated twice). Methanol extracts were then collected and stored at −80 °C until LC-MS/MS analysis. Following extraction, cells fixed on plates with methanol were used to determine protein content (surrogate for cell density) with sulforhodamine B (SRB) assay.

For protein determination, after the 48 h medium was discarded and cells were mechanically detached after addition of phosphate buffered saline (PBS) with protease inhibitor cocktail (Complete Tablets EDTA-free; Roche Diagnostics, Mannheim, Germany). Obtained cell suspensions were kept frozen at −80 °C until analysis.

### 4.4. Synthesis of the Novel Oxicam Drugs

Synthesis and experimental data of studied compounds #1–5 were previously reported [80,81,82]. Briefly, the starting material for the synthesis of the above-mentioned compounds was commercially available 1,1-dioxo-1,2-benzothiazol-3-one (saccharin). It was condensed with 2-bromoacetophenone (for compound #1) or 2-bromo-4′-fluoroacetophenone (for compounds #2, #3 and #4) or 2-bromo-4′-chloroacetophenone (for compound #5) in dimethylformamide (DMF) in the presence of triethylamine (TEA). The condensation products obtained were then rearranged to the corresponding 1,2-benzothiazine ring in Gabriel-Colman rearrangement. The final compounds were prepared by alkylation of corresponding 1,2-benzothiazine with 1-(3-chloropropyl)-4-phenylpiperazine (for compound #1 and #2) or 1-(3-chloropropyl)-4-(2-fluorophenyl)piperazine (for compound #3) or 1-(2-chloroacetyl)-4-(2-fluorophenyl)piperazine (for compounds #4 and #5) giving five new compounds. The separated products were purified by the crystallization from ethanol. The structures of the compounds obtained were confirmed by elemental and spectral analyses (MS, FTIR, 1H NMR, 13C NMR). Piroxicam (Sigma-Aldrich) and meloxicam (Alfa Aesar) as reference standards were obtained from commercial sources.

### 4.5. Analytical Methods

#### 4.5.1. Transcriptomic Analysis

##### RNA Extraction and Purification

Tissue fragments of 30–40 mg were homogenized in lysis buffer with 2-mercaptoethanol (100:1) (Sigma-Aldrich) using Fastprep 24 Homogenizer (MP Biomedical, Solon, OH, USA). 

Phenol-chloroform extraction was used for RNA isolation. Crude RNA was further purified using PureLink™ RNA Mini Kit (Invitrogen). On-column removal of genomic DNA with DNase (PureLink™ DNase Set, Invitrogen) was applied. Isolated RNA was quantified spectrophotometrically using NanoDrop 2000 from Thermo-Fisher Scientific. The purity of RNA isolates was determined as absorbance ratios of 260/280 nm and 260/230 nm. In turn, RNA integrity was evaluated using the LabChip microfluidic technology on Experion platform, using dedicated Experion RNA StdSens analysis kits (BioRad, Herkules, CA, USA).

##### Reverse Transcription

RNA, 500 ng (clinical samples) or 1000 ng (cell culture samples) per 20 µl of reaction mixture, was transcribed into cDNA using C1000 termocycler (BioRad) and iScript™ cDNA Synthesis Kit (BioRad) following suggested protocol.

##### Real-Time (Quantitative) PCR

qPCRs were conducted using SsoFast EvaGreen^®^ Supermix (BioRad) on CFX96 Real-Time PCR thermocycler (BioRad). The following cycling conditions were applied: activation at 95 °C for 30 s, denaturation at 95 °C for 5 s, annealing/extension at 61 °C for 5 s; 45 cycles, followed by melting curve analysis (60–95 °C with fluorescent reading every 0.5 °C). The reaction mixture consisted of cDNA (2 µL; diluted 1:5), 2 × SsoFast EvaGreen^®^ Supermix (10 µL), 10 nM forward and reverse target-specific primers (1 µL of each), and water up to 20 µL. Primers were synthesized by Genomed (Warsaw, Poland) and their characteristics is given in Table 4.

##### Normalization Strategy

Prior to analysis, technical replicates were averaged. For each sample set investigated, a geometric mean of all Cq values was calculated. It was subtracted from individual sample Cq, yielding ΔCq, subsequently linearized by 2^^ΔCq^ transformation, and normalized using geometric mean of *PPIA* and *RPLP0* for clinical samples [83] or expression of *GAPDH* for cell culture experiments. The obtained “normalized relative quantity” (NRQ) values [84] were then subjected to statistical analysis.

#### 4.5.2. Liquid Chromatography Coupled with Tandem Mass Spectrometry (LC-MS/MS)

Metabolite concentration in cell cultures was quantified using a method recently developed [85] and routinely used in our laboratory [6,7,86,87,88] with small modifications. 

##### Sample Preparation

Samples were dried using centrifugal vacuum concentrator (HETOVAC) at 50 °C and then re-dissolved in 10 µL of borate buffer (pH = 9.2), consisting of 0.025 M Na_2_B_4_O_7_ × 10H_2_O with 1.77 mM NaOH and 10 µL of internal standard solution (7 μM D6-ornithine, 10 μM D7-arginine, 0.1 μM D7-ADMA, 2 μM D4-cytrulline and 0.25 μM D6-DMA). After mixing for 1 min at 1200 rpm in 25 °C, 20 µL of acetonitrile with 10 µL of 10% benzoyl chloride in acetonitrile was added and mixed again. Aliquots (50 µL) were transferred into chromatographic glass vials with 0.2 mL borosilicate glass insert.

##### Chromatographic and Mass Spectrometry Analysis

Chromatographic analysis was performed using Acquity UPLC system (Waters, Milford, MA, USA). Sample temperature was set at 5 °C and column temperature at 60 °C. Samples (2 µL) were injected onto the Waters HSS T3 column (1.8 µm, 1.0 × 50 mm). The flow rate was 0.220 mL/min and a total run time was 8.50 min. The following gradient was used: 0.0 min—5% B hold for 1 min, 3.5 min—14% B, 5.0 min—60% B, 5.5 min—90% B hold for 1.1 min, 6.60 min—5% B, where eluent A was water with 0.1% formic acid (FA) and eluent B was methanol with 0.1% FA. 

Measurements were performed using Xevo G2 Q-TOF MS (Waters, Milford, MA, USA) with electrospray ionization (ESI) in positive ionization mode. The spray voltage, source temperature, and the desolvation temperature were set at 0.5 kV, 120 °C and 400 °C, respectively. Data were acquired using MassLynx software (Waters) for the following ions (m/z): 237.1239 (for ornithine), 243.1339 (for D6-ornithine), 279.1457 (for arginine), 286.1897 (for D7-arginine), 307.1770 (for ADMA and SDMA), 314.2209 (for D7-ADMA), 263.1090 (for citrulline), 267.1382 (for D4-cytrulline), 150.0919 (for DMA), and 156.1295 (D6-DMA). The following concentration ranges were prepared for standard calibration curves: 0.3–15 μM for ornithine, 0.5–25 μM for arginine, 0.005–0.25 μM for ADMA and SDMA, 0.1–5 μM for citrulline, and 0.014–0.7 μM for DMA.

Obtained metabolite concentrations were then normalized to cell density determined indirectly using the SRB assay.

##### Sulforhodamine B (SRB) Assay

The SRB assay allows for determining protein content, proportional to cell number. It was used to normalize the metabolomic results.

Solution of 0.04% SRB in 1% acetic acid (Sigma-Aldrich) was added to fixed cells and let stand for 30 min. Subsequently, the unbound dye was removed using 1% acetic acid. The protein-bound SRB was in turn solubilized by 10 mM Tris base solution (pH = 10.5). The absorbance, proportional to protein content, was measured using an Infinite M200 plate spectrophotometer (Tecan Group Ltd., Männedorf, Switzerland) at λ = 492 nm or 520 nm.

#### 4.5.3. Nitrate/Nitrite Determination

Nitrate/nitrite concentration as a surrogate for NO production was determined in conditioned media using Total Nitric Oxide and Nitrate/Nitrite assay (R&D Systems, Minneapolis, MN, USA) according to manufacturer’s instruction. Obtained concentrations of total and endogenous nitrite and nitrate were normalized to protein concentration in the media. Protein concentration was determined colorimetrically at λ = 595 nm (Infinite M200 plate spectrophotometer) using Bradford method against bovine serum albumin as a standard (Sigma).

#### 4.5.4. Determination of Protein Expression

Cell suspensions were thawed and sonicated on ice using the ultrasonic processor UP 200 (Hielscher Ultrasound Technology, Teltow, Germany), during two 30 sec cycles with amplitude set at 40%. To remove non-lysed cells, the suspension was centrifuged (12,500× *g*, 4 °C, 10 min.). Total protein concentration was measured colorimetrically at λ = 562 (EnSpire Multimode Plate Reader, PerkinElmer, Waltham, MA, USA) in the supernatants using the bicinchoninic acid (Thermo Fisher Scientific, Walthan, MA, USA) assay [89]. Protein samples (10 µg) were diluted 1:1 with 2 × Laemmli sample buffer (Bio-Rad, Hercules, CA, USA) containing 5% 2-mercaptoethanol, denatured (5 min at 95 °C), and resolved by SDS-PAGE electrophoresis [90]. Gels (0.75 mm, 4% stacking gel and a 10 % resolving gel) were run using Mini-Protean Tetra Cell (Bio-Rad) at constant voltage 200 V until the dye front reached the bottom of the gel. Subsequently, the separated proteins were transferred (30 min at constant voltage of 25 V) to the Immobilon P membrane (Merck-Millipore, Burlington, MA, USA). Dedicated transfer buffer (25 mM Tris, 190 mM glycine, 20% methanol) and Trans-Blot Turbo transfer system (Bio-Rad) were used. After the transfer, membranes were stained for total protein content using No-Stain™ Protein Labeling Reagent (Invitrogen, Carlsbad, CA, USA), according to manufacturer’s instructions. Then membranes were blocked with 1% casein blocking buffer (Sigma-Aldrich, Saint Louis, MO, USA) at room temperature for 1 h. After washing, the membranes were incubated (overnight, at 4 °C) with specific primary antibodies (Cusabio, Houston, TX, USA). The following dilutions were applied: arginase-2 (1:1000; cat. no. CSB-PA002006GA01HU), DDAH1 (1:1000; cat. no. CSB-PA877002), DDAH2 (1:5000; cat. no. CSB-PA006580LA01HU), PRMT5 (1:1000; cat. no. CSB-PA018734GA01HU), and PRMT1 (1:80; R&D Systems, Minneapolis, MN, USA; cat. No. AF6016). Antibodies were diluted in PBS-T buffer pH 7.3 (VWR, International, Radmor, PA, USA) with 0.05% Tween-20 (Thermo Fisher Scientific, Walthan, MA, USA). Unbound antibodies were removed by washing (3 times, 5 min) with PBS-T buffer. Then, the membranes were incubated with HRP-conjugated goat anti-rabbit antibodies (Invitrogen, Carlsbad, CA, USA; cat. no. A27036), diluted 1:50,000, or rabbit anti-goat antibodies (Sigma-Aldrich, Sain Louis, Mo, USA; cat. no. A5420), diluted 1:25,000. The analyzed proteins were visualized using Clarity Western ECL chemiluminescent substrate (Bio-Rad) and ChemiDoc MP Imaging System (Bio-Rad). The obtained Western-blot membrane images were analyzed using Image Lab software version 6.0.1 build 34 (Bio-Rad). The chemiluminescence intensity was normalized to the total protein intensity recorded for individual lanes.

### 4.6. Statistical Analysis

Normality of data distribution was tested using Kolmogorov-Smirnov test and homogeneity of variances using Levene test. Paired samples were examined using t-test for paired samples, on log-transformed data, if necessary. Independent samples were analyzed with t-test for independent samples or Mann–Whitney U test for two-group comparisons. One-way analysis of variance (ANOVA), with Tukey–Kramer post-hoc test, or Kruskal–Wallis H-test, with Conover post-hoc test, were applied for multi-group comparisons. Data are reported as means or medians with 95% confidence interval (CI). Spearman rank correlation test (*ρ*) was used to assess the association with cancer pathology. All applied tests were two-sided. *p* values < 0.05 were considered statistically significant. MedCalc Statistical Software version 19.4.0 (MedCalc Software Ltd., Ostend, Belgium; https://www.medcalc.org; 2020) was used for all analyses.

## 5. Conclusions

Metabolic reprogramming in CRC regarding L-arginine/NO pathway includes overexpression of dimethylarginine dimethylaminohydrolases (*DDAH1* and *DDAH2*) and protein methyltransferases (*PRMT1* and *PRMT5*), in addition to *ARG1* and *NOS2*. Importantly, transcriptional pattern of the pathway enzymes is altered not only in tumors, but also, or even only, in non-transformed tumor-adjacent tissue, contributing to the phenomenon of tumor molecular margin.

Novel oxicam analogues with reduced gastrotoxicity, differing from piroxicam and meloxicam with arylpiperazine moiety at the thiazine ring, are able to downregulate *DDAHs* and *PRMTs*, while classic oxicams had either weak or no effect on their expression. The presence of propylene linker between thiazine and piperazine nitrogens and two fluorine substituents, at arylpiperazine and benzoyl moieties, characterizes the most effective inhibitor of *DDAHs* and *PRMTs* expression. In addition to beneficial downregulation of *DDAHs* and *PRMTs* and ADMA accumulation, novel oxicam analogues upregulated *ARG2* expression and led to the potentially disadvantageous accumulation of arginine and SDMA. Further studies are needed to unravel the mechanisms by which novel oxicam analogues affect L-arginine/NO pathway. Those should include cell lines with varying and determined stage of carcinogenesis and encompass evaluation of analogues’ effect on amino acid transporters and other pathway enzymes.

## Figures and Tables

**Table 1:** Effect of novel oxicam analogues on arginine/nitric oxide pathway enzymes.

Gene	Comp.	5 µM			50 µM			200 µM		
		Caco2	HCT	HT29	Caco2	HCT	HT29	Caco2	HCT	HT29
***ARG2***	#1	↓(1.1)	↓1.2 ^2^	=	=	=	↑3.8 ^1^	↑1.8 ^1^	↑3.9 ^2^	↑7.0 ^2^
	#2	=	=	=	↓1.3 ^1^	=	↑6.7 ^1^	=	↑2.1 ^1^	↑6.4 ^2^
	#3	=	↓(1.3)	↑1.5 ^2^	=	=	↑4.0 ^1^	=	↑2.0 ^1^	↑(4.3)
	#4	=	=	=	↓1.2 ^1^	=	=	↑3.9 ^1^	=	↑3.8 ^2^
	#5	=	=	↓(1.6)	↓(1.4)	=	=	=	=	↑(7.0)
	P	=	=	=	=	=	=	=	=	=
	M	=	↑1.2 ^1^	=	=	=	=	=	↓2.0 ^2^	=
*DDAH1*	#1	=	=	=	=	=	↓1.9 ^1^	=	=	↓1.9 ^1^
	#2	=	=	=	↓1.7 ^1^	↓2.0 ^1^	=	=	↓1.7 ^2^	↓2.4 ^1^
	#3	=	=	↑(1.3)	=	↓(1.6)	↓(2.3)	=	↓2.2 ^1^	↓3.7 ^1^
	#4	=	=	=	=	=	=	=	=	=
	#5	=	=	=	=	=	=	=	=	↓3.0 ^1^
	P	=	=	=	=	=	=	=	=	=
	M	=	=	↓1.2 ^1^	=	=	=	=	=	↓(1.3)
*DDAH2*	#1	=	↓1.1 ^1^	=	=	=	↓1.5 ^1^	↓1.4 ^1^	=	↓(1.9)
	#2	=	↓1.5 ^1^	=	=	=	=	=	=	↓2.1 ^1^
	#3	=	=	↑1.4 ^2^	=	=	=	=	=	↓(3.0)
	#4	=	↓1.2 ^1^	=	=	↓1.6 ^1^	=	=	=	=
	#5	=	=	↓(1.3)	=	=	=	=	=	↓1.5 ^1^
	P	=	=	=	=	=	=	=	=	=
	M	=	=	=	=	=	=	=	=	↓1.8 ^1^
*NOS2*	#1	=	-	-	=	-	-	↑2.2 ^1^	-	-
	#2	=	-	-	↓(1.7)	-	-	↑3.3 ^2^	-	-
	#3	=	-	-	=	-	-	=	-	-
	#4	=	-	-	=	-	-	↑2.9 ^1^	-	-
	#5	=	-	-	=	-	-	↑(1.9)	-	-
	P	=	-	-	=	-	-	=	-	-
	M	=	-	-	=	-	-	=	-	-
*PRMT1*	#1	=	=	↑1.8 ^2^	=	=	=	=	=	↓(1.5)
	#2	=	=	=	↓(1.7)	↓2.1 ^1^	↓(1.6)	=	↓2.3 ^2^	↓2.4 ^2^
	#3	=	=	=	=	↓1.8 ^1^	↓2.1 ^2^	=	↓3.3 ^1^	↓4.6 ^2^
	#4	=	=	=	↓1.2 ^1^	=	=	=	=	↓(2.0)
	#5	=	=	=	=	=	=	=	=	↓3.2 ^1^
	P	=	=	=	=	=	=	=	=	=
	M	=	=	↓1.1 ^1^	=	=	=	=	=	↓(1.1)
*PRMT5*	#1	=	=	↑1.5 ^1^	=	↓(1.4)	=	↑1.6 ^2^	=	=
	#2	=	=	=	↓1.4 ^1^	↓2.1 ^1^	=	=	↓2.6 ^1^	↓1.7 ^1^
	#3	=	=	↑1.2 ^1^	↓1.5 ^1^	↓1.9 ^1^	=	=	↓3.6 ^2^	↓3.2 ^2^
	#4	=	=	=	=	=	=	=	↓3.8 ^1^	↓2.0 ^1^
	#5	=	=	↓(1.3)	=	=	=	=	=	↓2.4 ^1^
	P	↓(1.3)	=	=	=	=	=	=	=	=
	M	=	=	=	=	=	=	=	=	↓1.2 ^2^

Results of paired analysis showing a relative increase (↑) or decrease (↓) in gene expression normalized to *GAPDH* in treated as compared to non-treated cells (24-h incubation with indicated drug concentration). Data are presented as mean of three independent experiments and were analyzed using *t*-test for paired samples. Comp., compound; HCT, HCT 116 cell line; P, piroxicam; M, meloxicam; *ARG2*, arginase 2; *DDAH*, dimethylarginine dimethylaminohydrolase; *NOS2*, inducible nitric oxide synthase; *PRMT*, protein methyltransferase. ^1^ *p* < 0.05; ^2^ *p* < 0.01; =, no significant difference or tendency (*p* ≥ 0.1); - non-quantifiable. Tendencies (0.05 < *p* < 0.1) are given in brackets. Magnitude of relative change is shown in the form of a three-color-scaled heatmap (red-yellow-green, scaled from: −7.0 (dark red), through 1.0 (yellow) to 7.0 (dark green).

**Table 2:** Effect of Classic and Novel Oxicam Analogues on the Intracellular Level of Key Metabolites of Arginine/Nitric Oxide Pathway.

Gene	Comp.	5 µM			50 µM			200 µM		
		Caco2	HCT	HT29	Caco2	HCT	HT29	Caco2	HCT	HT29
**Arg**	#1	=	=	=	↑(1.4)	↑2.3 ^1^	↑2.8 ^1^	=	↑2.6 ^1^	↑4.2 ^1^
	#2	=	=	=	↑(1.5)	↑2.6 ^2^	↑3.4 ^1^	↑(2.2)	↑2.3 ^1^	↑2.9 ^1^
	#3	↑1.4 ^1^	=	=	↑(1.8)	↑2.1 ^1^	↑3.7 ^2^	↑1.3 ^2^	↑2.2 ^2^	↑(2.1)
	#4	↑(1.3)	=	↓(1.4)	↑1.4 ^2^	↑(1.6)	↑(2.0)	↑1.5 ^2^	=	↑3.3 ^1^
	#5	↑1.2 ^1^	=	↓(1.7)	↑(1.5)	=	=	↓2.2 ^1^	=	=
	P	↑1.4 ^1^	=	=	↑(1.5)	=	=	=	=	=
	M	↑1.5 ^2^	=	=	↑1.6 ^1^	=	=	=	=	=
Cit	#1	=	=	=	=	=	=	↑1.5 ^2^	=	=
	#2	=	=	↑1.3 ^1^	=	=	=	↑1.8 ^1^	↑2.1 ^1^	=
	#3	↑(1.1)	=	=	=	↑(2.0)	↑(3.2)	↑1.5 ^2^	↑2.2 ^1^	=
	#4	=	=	=	=	=	=	=	=	=
	#5	=	=	=	=	=	=	=	=	=
	P	=	=	=	=	=	=	=	=	=
	M	=	=	=	=	=	=	=	=	=
Orn	#1	↓(1.1)	=	=	=	=	↑(1.6)	↑2.3 ^2^	=	↑1.8 ^1^
	#2	=	=	=	=	↑2.7 ^1^	=	=	↑2.3 ^2^	↑(1.6)
	#3	↑1.1 ^1^	=	=	=	↑(2.2)	↑(1.6)	=	↑2.3 ^1^	=
	#4	=	=	=	=	=	=	=	=	=
	#5	=	=	=	=	↑(1.9)	=	=	↑(2.9)	↑(1.3)
	P	↑(1.1)	=	↓(1.1)	=	=	=	=	=	=
	M	=	↑2.3 ^1^	=	=	=	=	=	=	=
ADMA	#1	=	=	=	=	=	↑2.2 ^2^	↑1.3 ^1^	↑(1.8)	↑(2.3)
	#2	=	=	↑1.7 ^1^	=	↑(2.2)	↑(2.9)	↑1.7 ^2^	↑2.2 ^1^	=
	#3	=	=	=	=	=	↑(3.0)	↑1.5 ^1^	↑2.1 ^2^	=
	#4	=	=	=	=	=	=	↑(1.1)	=	=
	#5	=	=	=	=	=	=	=	=	=
	P	=	=	=	=	=	=	=	=	=
	M	=	=	=	=	=	=	=	=	↑1.7 ^1^
SDMA	#1	=	=	=	=	↑2.6 ^1^	↑2.6 ^1^	↑3.2 ^1^	↑2.5 ^1^	↑2.6 ^1^
	#2	=	=	=	=	↑2.4 ^1^	↑2.6 ^1^	↑2.0 ^1^	↑2.2 ^1^	↑2.3 ^1^
	#3	↑1.1 ^2^	=	=	↑(1.3)	=	↑2.6 ^1^	=	↑(1.9)	↑(1.8)
	#4	=	=	=	=	=	=	↑1.7 ^2^	=	↑2.0 ^1^
	#5	=	=	=	=	=	↑1.4 ^2^	=	=	=
	P	=	=	=	=	=	=	↑1.2 ^1^	=	=
	M	=	=	=	=	=	=	=	=	=
DMA	#1	=	=	=	=	=	=	=	=	↑(1.3)
	#2	=	=	=	↑(1.1)	↑(2.3)	=	=	=	=
	#3	=	=	=	=	↑(2.1)	=	=	↑(2.7)	=
	#4	=	=	=	↓1.1 ^1^	=	=	=	=	=
	#5	=	=	=	=	=	=	=	=	=
	P	↑(1.1)	=	=	=	=	↓2.1 ^1^	=	=	=
	M	=	=	↓2.9 ^1^	=	=	=	=	=	↓(1.8)

Results of paired analysis showing a relative increase (↑) or decrease (↓) in metabolite concentration normalized to protein content in treated as compared to non-treated cells (24-h incubation with indicated drug concentration). Data are presented as mean of three independent experiments and were analyzed using *t*-test for paired samples. Comp., compound; HCT, HCT 116 cell line; P, piroxicam; M, meloxicam; Arg, arginine; Cit, citrulline; Orn, ornithine; ADMA, asymmetric dimethylarginine; SDMA, symmetric dimethylarginine; DMA, dimethylamine; P, piroxicam; M, meloxicam. ^1^ *p* < 0.05; ^2^ *p* < 0.01; =, no significant difference or tendency (*p* ≥ 0.1); - non-quantifiable. Tendencies (0.05 < *p* < 0.1) are given in brackets. Magnitude of relative change is shown in the form of a three-color-scaled heatmap (red-yellow-green, scaled from: −7.0 (dark red), through 1.0 (yellow) to 7.0 (dark green).

**Table 3:** Patients’ characteristics.

Parameter	CRC
*N*	55
Sex (F/M), n	22/33
Age [yrs.], median (range)	68 (28–84)
Stage (0/I/II/III/IV)	8/6/14/23/4
Primary tumor, T (Tis/1/2/3/4)	8/2/7/30/8
Lymph node metastasis, N (0/1/2)	28/14/13
Distant metastasis, M (no/yes)	51/4
Grade, G (1/2/3/x)	8/36/7/4
Tumor location (left-side/right-side/rectum)	17/17/21

*N*, number of patients; F/M, female-to-male ratio; CRC, colorectal cancer; yrs., years; TNM, tumor-node-metastasis cancer staging system; T, depth of tumor invasion; Tis, tumor in situ; N, lymph node metastasis; M, distant metastasis; G, histological grade.

**Table 4:** Characteristics of applied primers.

Gene Symbol	Full Name	Accession No.	Sequence 5′→3′	Size [bp]
*PPIA* ^1^	Peptidylprolyl isomerase A	NM_021130.3	F: ggcaaatgctggacccaacaca R: tgctggtcttgccattcctgga	161
*RPLP0* ^1^	Ribosomal protein, large, P0	NM_001002.3	F: tcacaacaagcataccaagaagc R: gtatccgatgtccacaatgtcaag	263
*ARG1* ^2^	Arginase-1	NM_001244438.2	F: tcatctgggtggatgctcacac R: gagaatcctggcacatcgggaa	130
*ARG2* ^1^	Arginase-2	NM_001172.4	F: ctggcttgatgaaaaggctctcc R: tgagcgtggattcactatcaggt	119
*NOS2* ^1^	Inducible nitric oxide synthase	NM_000625.4	F: gctctacacctccaatgtgacc R: ctgccgagatttgagcctcatg	136
*PRMT1* ^1^	Arginine *N*-methyltransferase-1	NM_001536.5	F: tgcggtgaagatcgtcaaagcc R: ggactcgtagaagaggcagtag	142
*PRMT5* ^1^	Arginine *N*-methyltransferase-5	NM_006109.5	F: ctagaccgagtaccagaagagg R: cagcatacagctttatccgccg	136
*DDAH1* ^1^	Dimethylarginine dimethylaminohydrolase-1	NM_012137.4	F: atgcagtctccacagtgccagt R: ttgtcgtagcggtggtcactca	151
*DDAH2* ^1^	Dimethylarginine dimethylaminohydrolase-2	NM_001303007.2	F: ctttcttcgtcctgggttgcct R: ctccagttctgagcaggacaca	136
*GAPDH* ^2^	Glyceraldehyde-3-phosphate dehydrogenase	NM_001256799.3	F: tagattattctctgatttggtcgtattgg R: gctcctggaagatggtgatgg	223

^1^, Sequences designed by Origene (www.origene.com); ^2^, sequences designed with Beacon Designer Probe/Primer Design Software (BioRad). All sequences were validated in silico (Blast analysis), and their specificity confirmed in melting curve analysis and agarose electrophoresis. F, forward primer; R, reverse primer.

## Supplementary Materials

The following are available online at https://www.mdpi.com/2072-6694/12/9/2594/s1, Figure S1: Structures of classic and new oxicam analogues, Figure S2: Pairwise (patient-matched) analysis of expression of arginine/nitric oxide pathway enzymes in patients with colorectal adenocarcinoma, Figure S3: Fold-change in expression of arginine/nitric oxide pathway enzymes in patients with colorectal adenocarcinomas in relation to cancer pathological features, Figure S4: Impact of oxicams on Caco-2 cell viability determined with sulforhodamine B assay, Figure S5: Impact of oxicams on HCT 116 cell viability determined with sulforhodamine B assay, Figure S6: Impact of oxicams on HT-29 cell viability determined with sulforhodamine B assay, Table S1: Effect of novel oxicam analogues on L-arginine/nitric oxide pathway enzymes, Figure S7: Western Blot analysis of protein expression level of ARG2, DDAH1, DDAH2, PRMT1, PRMT5 in HT-29.
