# Supplementary material for: L-Arginine/Nitric Oxide Pathway Is Altered in Colorectal Cancer and Can Be Modulated by Novel Derivatives from Oxicam Class of Non-Steroidal Anti-Inflammatory Drugs
Source: Cancers (Basel). 2020 Sep 11;12(9):2594. doi: 10.3390/cancers12092594 (PMC7564351; doi:10.3390/cancers12092594)
Supplement: Supplementary file 1 [file cancers-12-02594-s001.pdf]

Supplementary Material; Krzystek-Korpacka et al.

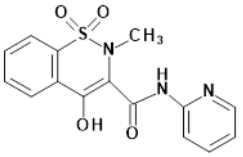

Piroxicam

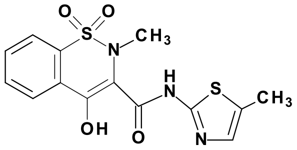

meloxicam

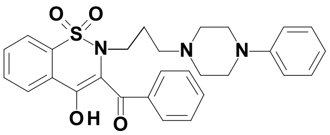

compound #1

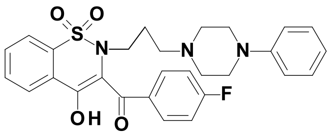

compound #2

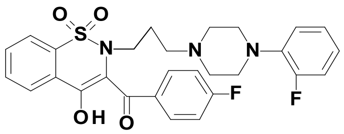

compound #3

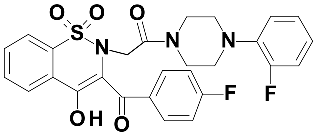

compound #4

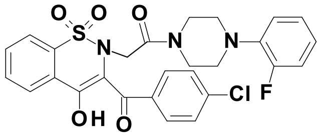

compound #5

Figure S1. Structures of classic and new oxicam analogues.

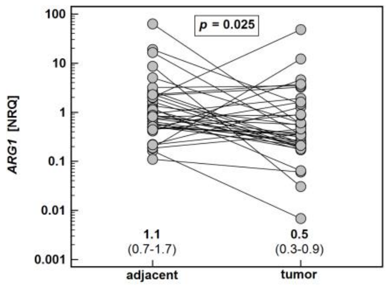

(a)

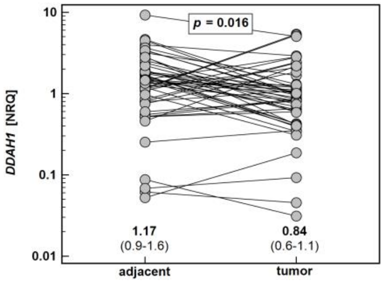

(b)

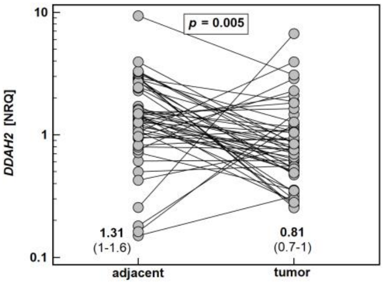

(c)

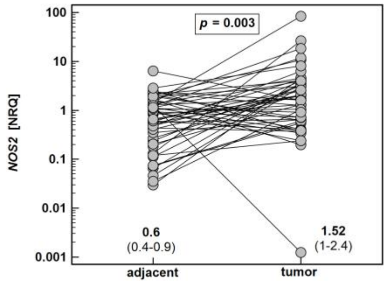

(d)

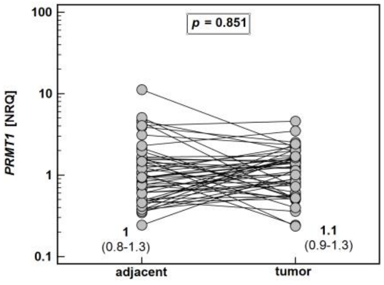

(e)

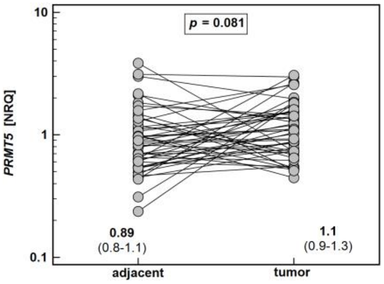

(f)

Figure S2. Pairwise (patient-matched) analysis of expression of arginine/nitric oxide pathway enzymes in patients with colorectal adenocarcinomas: (**a**) *ARG1*; (**b**) *DDAH1*; (**c**) *DDAH2*; (**d**) *NOS2*; (**e**) *PRMT1*; (**f**) *PRMT5*. Log-transformed data were analyzed using *t*-test for paired samples and additionally presented as geometric mean of normalized relative quantities (NRQ) with 95% confidence interval. *ARG2*, arginase 2; *DDAH*, dimethylarginine dimethylaminohydrolase; *NOS2*, inducible nitric oxide synthase; *PRMT*, protein methyltransferase.

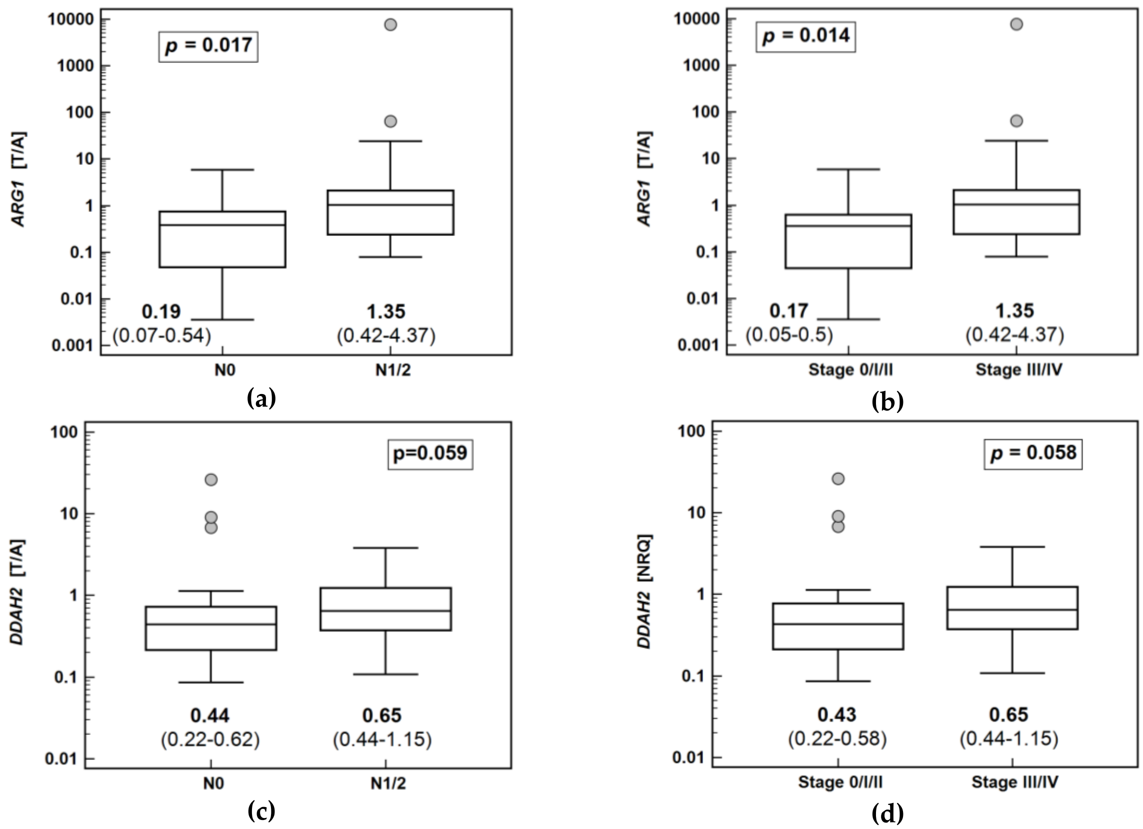

Figure S3. Fold-change (tumor-to-adjacent ratio; T/A) in expression of arginine/nitric oxide pathway enzymes in patients with colorectal adenocarcinomas in relation to cancer pathological features: (**a**) *ARG1* association with lymph node metastasis (N); (**b**) *ARG1* association with cancer stage; (**c**) *DDAH2* association with lymph node metastasis; (**d**) *DDAH2* association with cancer stage. Data analyzed using *t*-test for independent samples (*ARG1*) or Mann-Whitney *U*-test (*DDAH2*) and numeric data resent, respectively, means or medians with 95% confidence interval. Boxes indicate interquartile range with median, whiskers – 95% confidence interval; grey dots – outlying observations. *ARG2*, arginase 2; *DDAH2*, dimethylarginine dimethylaminohydrolase 2.

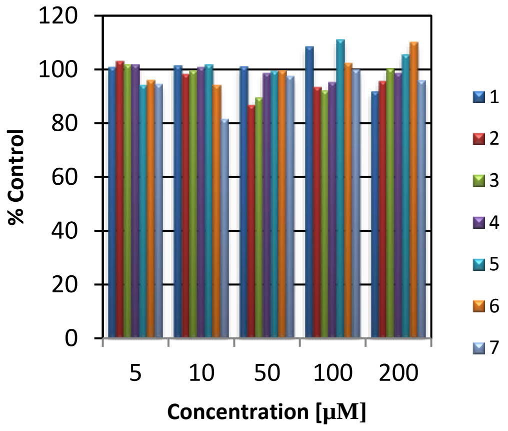

(a)

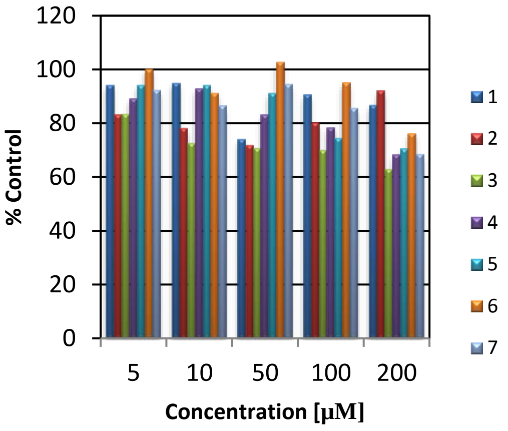

(b)

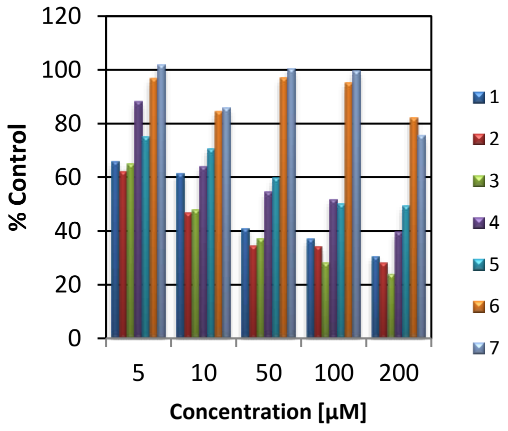

(c)

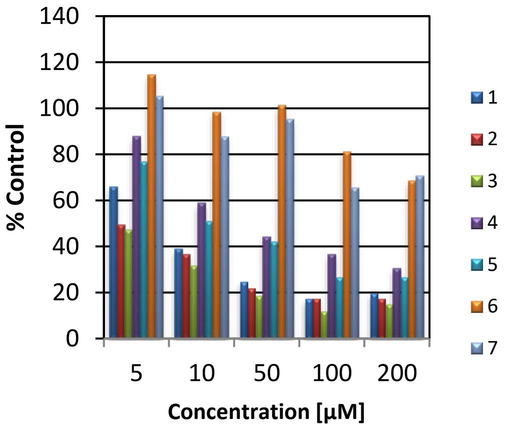

(d)

Figure S4. Impact of oxicams on Caco-2 cell viability determined with sulforhodamine B assay: (**a**) cells treated for 6 hours; (**b**) cells treated for 24 hours; (**c**) cells treated for 48 hours; (**d**) cells treated for 72 hours. Compounds #1-5 (novel oxicam analogues) marked as numbers 1-5, piroxicam marked as 6 and meloxicam marked as 7. Data presented as percentage (% control) of viability of untreated cells.

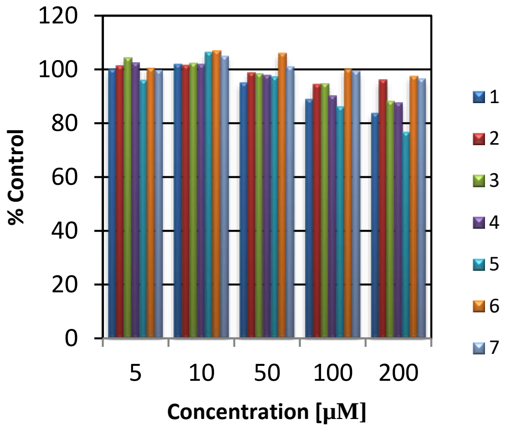

(a)

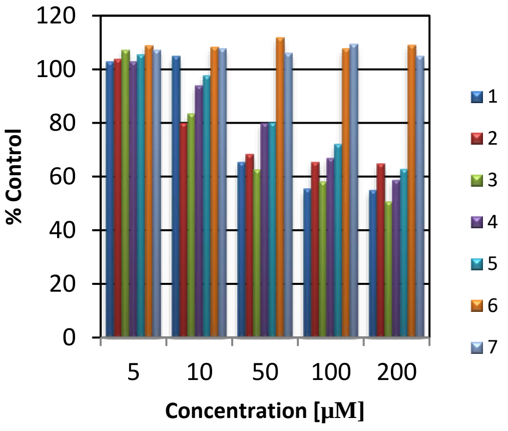

(b)

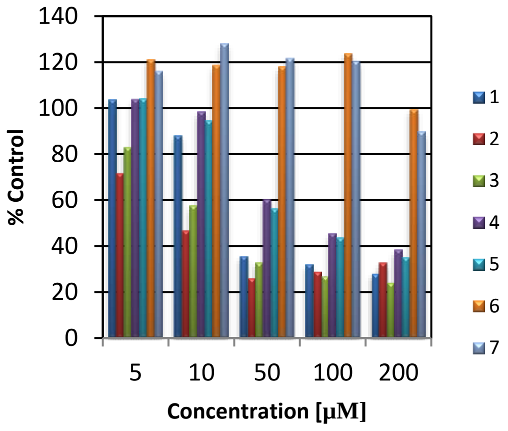

(c)

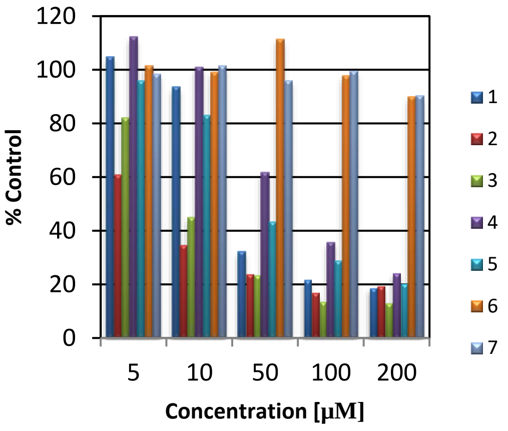

(d)

Figure S5. Impact of oxicams on HCT 116 cell viability determined with sulforhodamine B assay: (**a**) cells treated for 6 hours; (**b**) cells treated for 24 hours; (**c**) cells treated for 48 hours; (**d**) cells treated for 72 hours. Compounds #1-5 (novel oxicam analogues) marked as numbers 1-5, piroxicam marked as 6 and meloxicam marked as 7. Data presented as percentage (% control) of viability of untreated cells.

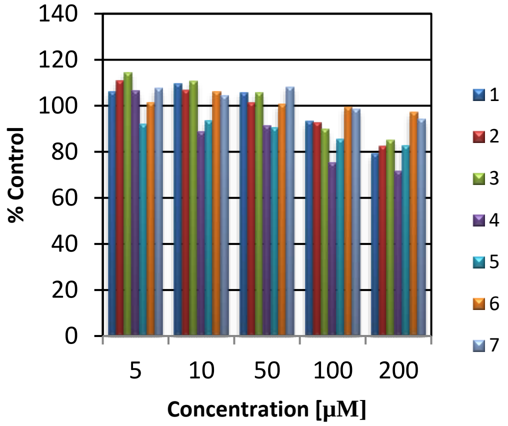

(a)

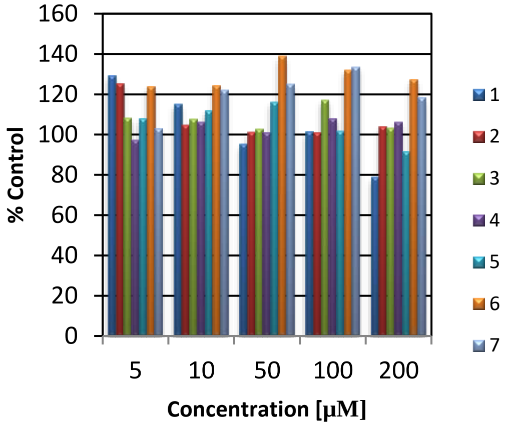

(b)

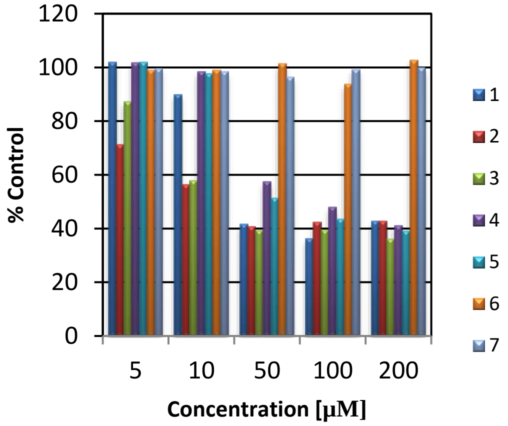

(c)

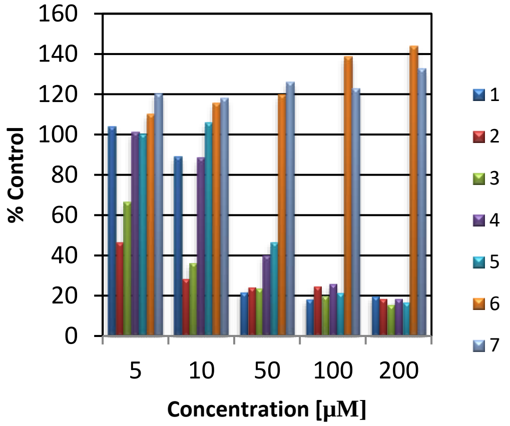

(d)

Figure S6. Impact of oxicams on HT-29 cell viability determined with sulforhodamine B assay: (**a**) cells treated for 6 hours; (**b**) cells treated for 24 hours; (**c**) cells treated for 48 hours; (**d**) cells treated for 72 hours. Compounds #1-5 (novel oxicam analogues) marked as numbers 1-5, piroxicam marked as 6 and meloxicam marked as 7. Data presented as percentage (% control) of viability of untreated cells.

Table S1. Effect of novel oxicam analogues on L-arginine/nitric oxide pathway enzymes

Gene	Comp.	5 μM 72h			200 μM 6h		
		Caco-2	HCT-116	HT 29	Caco-2	HCT-116	HT 29
ARG2	#1	-	-	-	=	↑1.6[1]	↑(2.5)
	#2	-	-	-	=	=	↑(3.4)
	#3	-	-	-	=	=	↑3.5[1]
	#4	=	↓1.3[1]	=	=	↑(1.4)	=
	#5	=	=	=	=	=	↑2.6[1]
	P	=	=	=	=	=	=
	M	=	↓1.5[1]	↓(2.3)	=	=	=
DDAH1	#1	-	-	-	=	=	=
	#2	-	-	-	=	=	=
	#3	-	-	-	=	=	↓1.3[1]
	#4	=	=	=	=	↓(1.3)	=
	#5	=	=	=	=	↓(1.4)	=
	P	=	=	=	=	=	=
	M	=	=	=	=	=	↑1.1[1]
DDAH2	#1	-	-	-	=	=	=
	#2	-	-	-	↓(1.9)	=	=
	#3	-	-	-	=	=	=
	#4	=	=	=	=	↓1.5[1]	(↓1.4)
	#5	=	=	=	=	=	=
	P	=	=	=	=	=	=
	M	=	↓1.3[2]	↓(1.6)	=	=	=
NOS2	#1	-	-	-	=	-	-
	#2	-	-	-	↓(2.3)	-	-
	#3	-	-	-	=	-	-
	#4	=	-	-	=	-	-
	#5	=	-	-	=	-	-
	P	=	-	-	=	-	-
	M	=	-	-	=	-	-
PRMT1	#1	-	-	-	=	=	=
	#2	-	-	-	=	=	=
	#3	-	-	-	=	=	↓1.3[1]
	#4	=	=	=	↓1.2[1]	=	=
	#5	=	=	=	↓1.3[1]	=	=
	P	=	=	=	↓(1.3)	=	=
	M	=	=	=	=	=	=
PRMT5	#1	-	-	-	=	=	=
	#2	-	-	-	=	=	=
	#3	-	-	-	=	=	↓(1.1)
	#4	↓1.1[1]	↓(1.4)	↓(1.1)	=	↓1.3[1]	=
	#5	=	=	=	=	=	=
	P	↓(1.2)	=	=	=	=	=
	M	=	↓1.4[1]	=	=	=	↑(1.2)

Results of paired analysis showing a fold increase (↑) or decrease (↓) in gene expression normalized to *GAPDH* in treated as compared to non-treated cells (72-hour incubation with 5 μM drugs or 6-hour incubation with 200 5 μM drugs). Data present mean of three independent experiments and were analyzed using *t*-test for paired samples. Comp., compound; P, piroxicam; M, meloxicam; *ARG2*, arginase 2; *DDAH*, dimethylarginine dimethylaminohydrolase; *NOS2*, inducible nitric oxide synthase; *PRMT*, protein methyltransferases. [1] $p < 0.05$; [2] $p < 0.01$; =, no significant difference or tendency ($p \geq 0.1$); - non-quantifiable. Tendencies ($0.05 < p < 0.1$) are given in brackets.

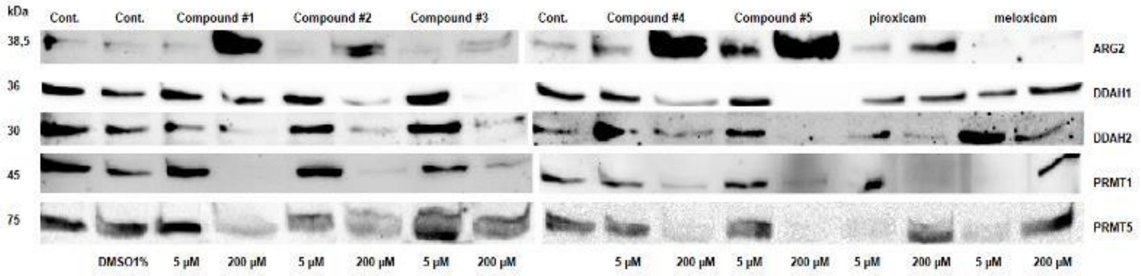

Figure S7. Western Blot analysis of protein expression level of ARG2, DDAH1, DDAH2, PRMT1, PRMT5 in HT-29. Cells were treated with the indicated concentrations (5 or 200 μM) of compounds #1-5, piroxicam, and meloxicam for 48 hours. The blots were processed and cropped using Image Lab software. Cont., untreated cells (incubated with 1% dimethyl sulfoxide (DMSO) in case of compound #1); ARG2, arginase 2; DDAH, dimethylarginine dimethylaminohydrolase; PRMT, protein methyltransferase.

Table S2. Effect of classic and novel oxicam analogues on intracellular levels of key metabolites of L-arginine/NO pathway

Metabolite	Comp.	5 μM 72h			200 μM 6h		
		**Caco-2**	**HCT-116**	**HT 29**	**Caco-2**	**HCT-116**	**HT 29**
Arg	#1	-	-	-	-	↑ (1.1)	↑1.8[1]
	#2	-		-	-	=	=
	#3			-	-	=	=
	#4	=	↓(1.2)	=	-	=	=
	#5	=	=	=	-	=	=
	P	=	=	=	-	=	=
	M	=	=	=	-	=	=
Cit	#1	-	-	-	-	=	=
	#2	-	-	-	-	↑ (1.1)	=
	#3	-	-	-	-	=	=
	#4	↑ (1.1)	↓ 1.1[2]	=	-	=	=
	#5	↑ 1.1[2]	=	=	-	=	=
	P	=	=	=	-	=	=
	M	=	=	=	-	=	=
ADMA	#1	-	-	-	-	=	↑1.6[1]
	#2	-	-	-	-	↑ 1.1[1]	=
	#3	-	-	-	-	=	=
	#4	=	=	=	-	=	=
	#5	=	=	=	-	=	=
	P	=	=	=	-	=	=
	M	=	=	=	-	=	
SDMA	#1	-	-	-	-	=	↑1.8[1]
	#2	-	-	-	-	=	=
	#3	-	-	-	-	=	=
	#4	=	↓ (1.3)	=	-	=	=
	#5	=	=	=	-	=	=
	P	=	=	=	-	=	=
	M	=	=	=	-	=	=
DMA	#1	-	-	-	-	=	=
	#2	-	-	-	-	=	=
	#3	-	-	-	-	=	=
	#4	=	=	=	-	↓ (1.1)	=
	#5	↓ (1.2)	=	=	-	=	=
	P	=	=	=	-	=	=
	M	=	=	=	-	=	=
Orn	#1	-	-	-	-	=	↑1.1[1]
	#2	-	-	-	-	=	=
	#3	-	-	-	-	=	=
	#4	↑	↓ 1.3[1]	=	-	=	=
	#5	↑ (1.1)	=	=	-	=	=
	P	=	=	=	-	=	=
	M	=	=	=	-	=	=

Results of paired analysis showing a fold increase (↑) or decrease (↓) in metabolite concentration adjusted to protein content in treated as compared to non-treated cells (72-hour incubation with 5 μM drugs or 6-hour incubation with 200 5 μM drugs). Data present mean of three independent experiments and were analyzed using *t*-test for paired samples. Comp., compound; Arg, arginine; Cit, citrulline; ADMA, asymmetric dimethylarginine; SDMA, symmetric dimethylarginine; DMA, dimethylamine; Orn, ornithine; P, piroxicam; M, meloxicam; [1] $p < 0.05$; [2] $p < 0.01$; =, no significant difference or tendency ($p \geq 0.1$). Tendencies ($0.05 < p < 0.1$) are given in brackets.
